# *Arabidopsis thaliana* PrimPol is a primase and lesion bypass DNA polymerase with the biochemical characteristics to cope with DNA damage in the nucleus, mitochondria, and chloroplast

**DOI:** 10.1038/s41598-021-00151-7

**Published:** 2021-10-18

**Authors:** Paola L. García-Medel, Antolín Peralta-Castro, Noe Baruch-Torres, Alma Fuentes-Pascacio, José A. Pedroza-García, Alfredo Cruz-Ramirez, Luis G. Brieba

**Affiliations:** 1 Laboratorio Nacional de Genómica para la Biodiversidad, Centro de Investigación y de Estudios Avanzados del IPN, Apartado Postal 629, Km. 9.6 Libramiento Norte Carretera, Irapuato-León, CP 36821 Irapuato, Guanajuato Mexico; 2 Departamento de Biología Molecular de Plantas, Instituto de Biotecnología, Universidad Nacional Autónoma de México (UNAM), Apartado Postal 510-3, 62250 Cuernavaca, Morelos Mexico; 3 Present Address: Department of Pharmacology and Toxicology, The University of Texas Medical Branch at Galveston, Galveston, TX 77555 USA

**Keywords:** DNA, Enzymes

## Abstract

PrimPol is a novel Primase–Polymerase that synthesizes RNA and DNA primers de novo* a*nd extents from these primers as a DNA polymerase. Animal PrimPol is involved in nuclear and mitochondrial DNA replication by virtue of its translesion DNA synthesis (TLS) and repriming activities. Here we report that the plant model *Arabidopsis thaliana* encodes a functional PrimPol (AtPrimPol). AtPrimPol is a low fidelity and a TLS polymerase capable to bypass DNA lesions, like thymine glycol and abasic sites, by incorporating directly across these lesions or by skipping them. AtPrimPol is also an efficient primase that preferentially recognizes the single-stranded 3′-GTCG-5′ DNA sequence, where the 3′-G is cryptic. AtPrimPol is the first DNA polymerase that localizes in three cellular compartments: nucleus, mitochondria, and chloroplast. In vitro, AtPrimPol synthesizes primers that are extended by the plant organellar DNA polymerases and this reaction is regulated by organellar single-stranded binding proteins. Given the constant exposure of plants to endogenous and exogenous DNA-damaging agents and the enzymatic capabilities of lesion bypass and re-priming of AtPrimPol, we postulate a predominant role of this enzyme in avoiding replication fork collapse in all three plant genomes, both as a primase and as a TLS polymerase.

## Introduction

Plants nuclear, mitochondrial, and plastid genomes are exposed to DNA damaging agents like ultraviolet light, ionizing radiation, and reactive oxidative species^1,2^. DNA polymerases (DNAP) stall at DNA lesions leading to replication fork collapse. In order to avoid stalled replication forks, nature has evolved DNAPs specialized in translesion DNA synthesis (TLS) that efficiently incorporate and extend from DNA lesions^3^. Flowering plants encode DNAPs specialized in TLS that are orthologous to human DNAPs ζ, η, θ, ι, and κ^4–10^ and they also harbor a unique family of DNAPs, dubbed plant organellar DNAPs (POPs), that are targeted to both mitochondria and chloroplast^11,12^. These polymerases execute TLS and microhomology-mediated end-joining (MMEJ)^13–17^ and are the sole DNAPs identified to date in plant organelles^11^.

Although DNAPs with TLS activities minimize replication fork collapse, an alternative mechanism for its avoidance is repriming downstream of a DNA lesion^18,19^. Besides nuclear replicative primases from the PriS/Prim1 family, eukaryotes bear a novel primase from the archaeo-eukaryotic primase (AEP) superfamily^20–23^. This enzyme harbors primase and TLS DNAP activities, and because of the presence of both enzymatic activities in a single polypeptide this enzyme is dubbed PrimPol^21–23^. The modular architecture of PrimPol harboring independent AEP and a Zn^++^ finger (Zf) domains allows recognition of single-stranded DNA templates for primer synthesis and extension^21,24–27^. During primer synthesis, the Zf domain interacts with the initial nucleotide and the cryptic G^27,28^ and structural studies shown that the AEP domain stablishes limited contacts with the primer-strand, in principle allowing the active site to bind two nucleotides simultaneously^26^. These two structural features of by PrimPols allow template recognition and priming initiation^28^. Thus, PrimPol is a TLS DNAP and primase implicated in avoiding replication fork collapse by its intrinsic TLS and by repriming downstream of the lesion^21–23^. Human PrimPol (HsPrimPol) in complex with a template/primer DNA and an incoming deoxynucleotide illustrates that the AEP domain of PrimPol makes limited contacts with the primer-strand allowing the active site to bind two nucleotides at the same time^26^ and biochemical studies demonstrate that the Zn-finger domain interacts with the initial nucleotide and the cryptic G. HsPrimPol is localized in the nucleus and mitochondria^21,24^. Although HsPrimPol is a non-essential protein, this enzyme is involved in repriming, even in the absence of damaged DNA, at lesions that block nuclear DNA or structures that halt the replication fork^22,29–32^.

In contrast to the well-studied HsPrimPol, little is known about this enzyme in other eukaryotes. Phylogenetic analyses indicated the presence of orthologous PrimPol genes in unicellular algae and land plants^20,21^. Here, we report that the PrimPol ortholog from the plant model *Arabidopsis thaliana* (AtPrimPol) is a translesion synthesis DNA polymerase and primase able to synthesize primers for DNA synthesis and that this enzyme is the sole DNA polymerase identified to date localized in three different cellular compartments: nucleus, mitochondria and chloroplast.

## Results

### The nuclear Arabidopsis genome encodes a PrimPol

**Figure 1:**
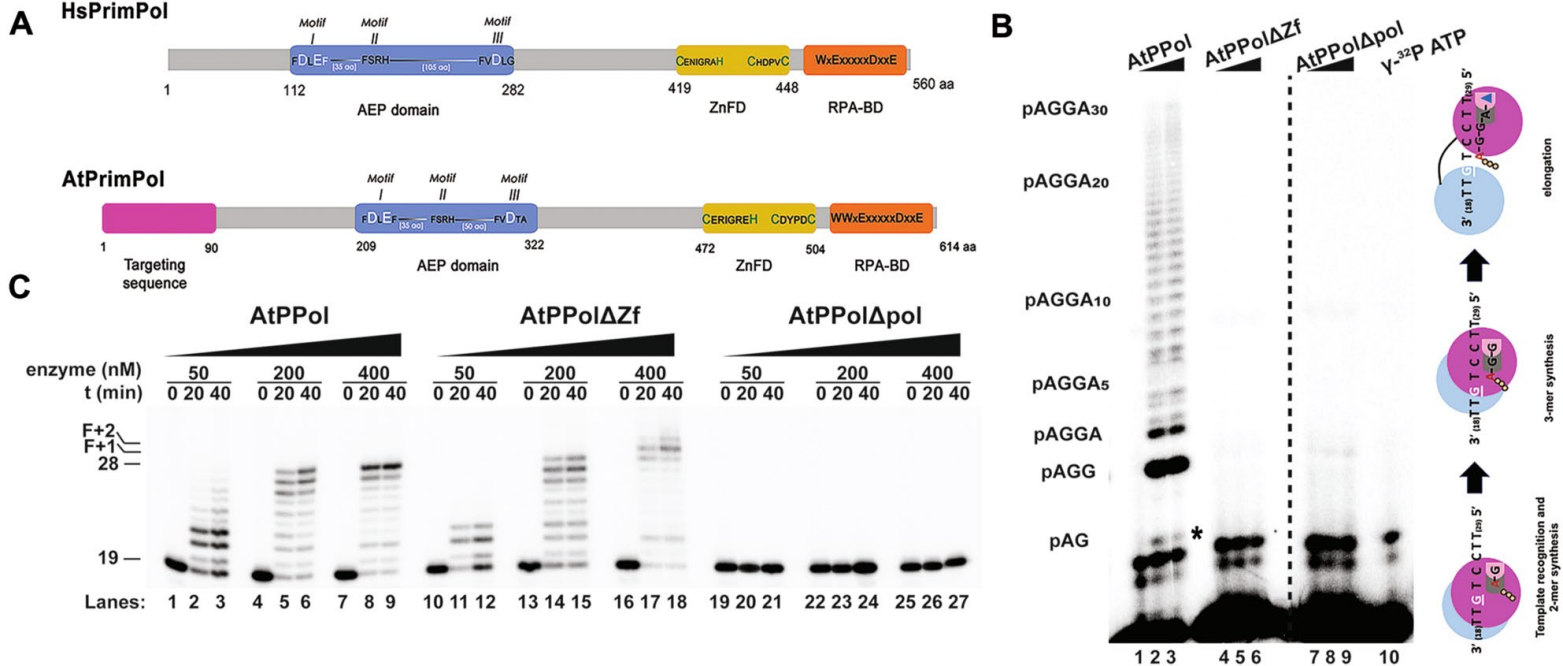
AtPrimPol (AtPPol) is an active DNA polymerase and template-dependent primase with conserved AEP structural organization. (**A**) Domain organization of AtPrimPol in comparison to human PrimPol. The conserved motifs in the AEP and Zn^++^ finger subdomains and the N-terminal targeting sequence are indicated. (**B**) Primer synthesis by AtPPol using rNTPs. Ribonucleotide synthesis by AtPPol (200 and 400 nM) on ssDNA template containing the recognition sequence 5′-CCTG. Primer synthesis is observed by AtPPol, while the mutant lacking the Zf domain (AtPPolΔZf) and a mutant of the DXE polymerization motif (AtPPolΔpol) are unable to carry out primer synthesis**.** Primer synthesis starts with a 5′-pAG-3′ dinucleotide that is elongated to an oligonucleotides of 33 base pairs (5′-pAGGA(_30_)-3′). The process of template recognition, dimer synthesis, and primer extension by AtPrimPol is depicted as a cartoon. The cyan sphere represents the Zn++ finger domain and the magenta sphere the APE domain. (**C**) AtPrimPol is an active DNA polymerase. Primer extension reaction using three concentrations of wild-type and variants of AtPrimPol. Primer extension is observed for AtPPol, and the PrimPol ∆ZnF mutant whereas the mutant in the DXE polymerization motif is inactive. Original data used to compose this figure is present in Fig. S8.

Bioinformatics analysis identified a gene orthologous to human PrimPol (HsPrimPol) in the model plant *A. thaliana*^20,21^. To further expand this analysis, the amino acid sequence of HsPrimPol was used as a bait to search the Arabidopsis Information Resource (TAIR) database^33^. A single hit corresponding to the *At5g52800* gene was retrieved from this search. Because of alternative splicing, it is predicted that the *At5g52800* gene encodes for three PRIMPOL isoforms of 614, 506, and 450 amino acids (Fig. 1A, Supplementary Fig. S1). All PRIMPOL isoforms of *A. thaliana* (AtPrimPol) carry a unique N-terminal 90 amino acid sequence not present in HsPrimPol (Fig. 1A, Supplementary Fig. S1). This N-terminal amino acid sequence encodes for potential transit peptides to chloroplast (residues 47 to 67) and mitochondria (residues 52 to 73) and two nuclear localization sequences^34^. An amino acid sequence alignment between AtPrimPol isoform 3 and HsPrimPol depicts the conservation in motifs I, II, and III of the AEP domain, which are involved in DNA polymerization, between both proteins (Fig. S2). AtPrimPol isoform 3 shares 34% amino acid identity with HsPrimPol (Fig. S1), and herein after this isoform is referred as AtPrimPol. AtPrimPol orthologues are present in all members of the kingdom Plantae, including the green algae *Micromonas pusilla* and Bryophytes like *Marchantia polymorpha* (Fig. S3), suggesting the presence of this gene before the divergence between plants and animals. Throughout the divergence between plant and animal PrimPols have conserved the motifs to interact with replication protein A (RPA) (Fig. S2), implying that this interaction is conserved in the plant kingdom^35^. Although AtPrimPol and HsPrimPol exhibit a similar domain architecture, these enzymes present two notable differences in their catalytic core: (1) the linker region between motifs II and III of the AEP domain is 50 amino acids shorter in AtPrimPol^26^, and (2) the interdomain region between the AEP and Zn^++^ finger binding domains is 18 amino acids longer in AtPrimPol (Fig. S2).

### AtPrimPol is a DNA polymerase and a DNA primase

To investigate the predicted DNA polymerase and primase activities of AtPrimPol, we use recombinantly expressed AtPrimPol lacking its putative organellar targeting peptide (residues 1 to 86) that we dubbed wild-type, a mutant that deletes its Zn^++^ finger subdomain (AtPPol∆Zf) (residues to 387–614), and a mutant defective in metal coordination at the AEP domain (D209A/E211A) (AtPPol∆pol) using heterologous expressed proteins (Supplementary Fig. S4A,B, S13). To investigate the putative primase activity of AtPrimPol, we used a single-stranded oligonucleotide that harbors the preferred template sequence for HsPrimPol, 3′-T_(15)_GTCCT_(30)_-5′, in which the underlined G is cryptic (necessary for recognition but not used as a template)^21^. In all enzymatic assays, we used a mixture of 10 mM MgCl_2_ and 1 mM MnCl_2_, as these are the optimal conditions to assay the DNA polymerase activity of AtPrimPol (Supplementary Figs. S4C, S13). We did not use Mn^++^ as a sole divalent cation because AtPrimPol recognizes alternative recognition nucleotide sequences under those conditions (data not shown).

**Figure 2:**
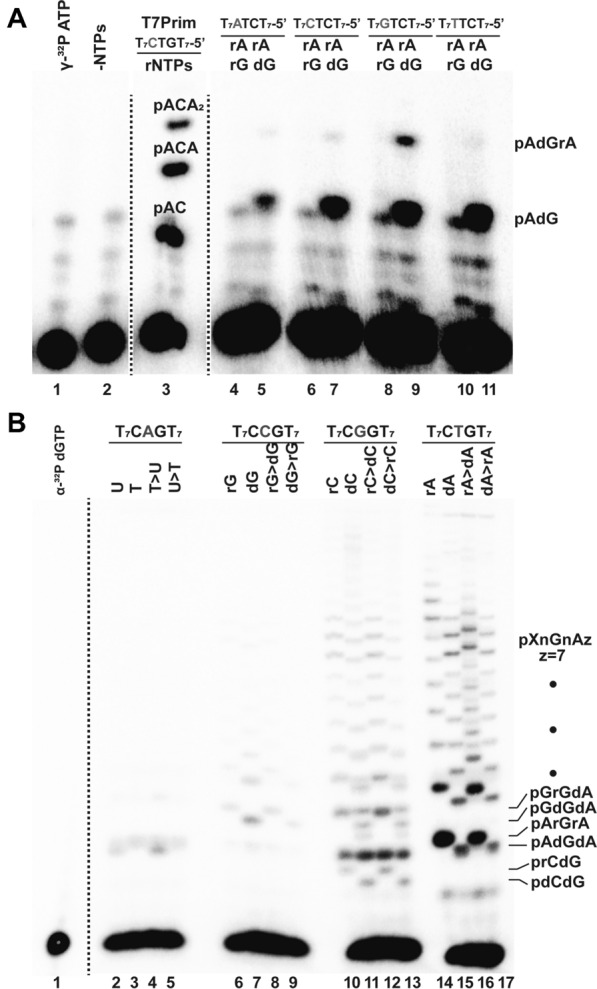
AtPrimPol recognizes a single-stranded DNA sequence and preferentially incorporates dNTPs over rNTPS. (**A**) AtPrimPol recognizes the cryptic G. (**A**) 24% denaturing gel electrophoresis showing AtPrimPol primer synthesis assayed on four different templates where the base corresponding to the cryptic nucleotide was systematically changed (lanes 4 to 11). Reactions were carried out in the presence of ATP and GTP or dGTP. Primer synthesis was mainly observed in reactions contained dGTP and longer products were only synthesized on a template containing the 3′-GTC-5′ motif. A control reaction showing primer synthesis (pAC and pACA products) by T7 Primase is used as molecular weight marker (lane 3). (**B**) AtPrimPol prefers rATP in its 5′ position. Primer formation was evaluated on four templates containing the 3′-GAC-5′, 3′-GCC-5′, 3′-GGC-5′, and 3′-GTC-5′ sequences. Each of these templates were assayed with U/T, G/dG, C/dC and A/dA respectively at 100 μM, and 100:1 μM. Primer synthesis on templates containing the CAG and CCG sequences was inefficient, whereas templates containing the 3′-GGC-5′ and 3′-GTC-5′ sequences readily primed and extended beyond the length of the template. Original data used to compose this figure is present in Fig. S9.

DNA primases from the DnaG and AEP superfamilies (i.e. bacterial DnaG and eukaryotic PriS/Prim1, respectively) synthesize short RNA primers for their cognate DNAPs^36^. In contrast PrimPol has a remarkable ability to synthesis DNA primers using dNTPs instead or NTPs^21,37^. Because of the traditional role of primases in synthetizing RNA primers, we decided to investigate if AtPrimPol was able to use solely NTPs as a substrate. In reactions labeled with [γ-^32^P] ATP, in the presence of GTP and ATP, full-length AtPrimPol is the sole construct able to synthesize ribo-oligonucleotides. AtPrimPol is able to synthesize primers of 33 ribo-oligonucleotides, that correspond to the maximum length of the template substrate starting from the thymidine adjacent to the cryptic guanosine (Fig. 1B, lanes 1 to 3, Fig. S8). Both AtPPol∆Zf and AtPPol∆pol mutants are unable to carry out primer synthesis indicating a prevalent role of the Zn^++^ finger in primer synthesis and the necessity of metal coordination for nucleotide incorporation (Fig. 1B, lanes 4 to 9, Fig. S8). To investigate DNA polymerization activity by AtPrimPol, we hybridized a 19-mer radiolabeled primer to a 28-mer template. At a 50-fold excess of AtPrimPol, this enzyme synthesizes products that reach the end of the template after an incubation period of 40 min (Fig. 1C, lanes 1 to 9, Fig. S8). AtPPol∆Zf efficiently performs template-dependent dNTP incorporation and at higher enzyme concentrations, this mutant performs the addition of one or two non-templated nucleotides (Fig. 1C, lanes 10 to 18, Fig. S8). As expected, the AtPPol∆pol mutant is unable to perform nucleotide incorporation^26^ (Fig. 1C, lanes 19 to 27, Fig. S8). HsPrimPol preferentially starts de novo primer synthesis on single-stranded templates that provide a cryptic guanosine^21,27^. In order to test if AtPrimPol selectively uses a cryptic guanosine during priming synthesis we used a template in which the identity of the cryptic 3′-base was systematically modified as follows: 3′-ATC-5′, 3′-CTC-5′, 3′-GTC-5′, and 3′-TTC-5′ (Fig. 2A, Fig. S9). In the presence of [γ-^32^P] ATP and dGTP or rGTP, AtPrimPol executes 3pAdG synthesis in all substrates and as is the case for all primases, the initial nucleotide harbors a triphosphate group on the or 5′ of the growing chain. However, AtPrimPol only efficiently synthetizes the trinucleotide 3pAdGrA on the 3′-GTC-5′ substrate, indicating a moderate preference for the cryptic guanosine (Fig. 2A, lanes 4 to 11).

In reactions harboring dGTP instead of rGTP (Fig. 2A, lanes 5, 7, 9, and 11), AtPrimPol substantially increases the amount of synthesized dinucleotides, indicating that as human PrimPol, AtPrimPol preferentially incorporates dNTPs instead of rNTPs^21,37^.

In order to investigate the preference for the initial 5′ nucleotide, we executed an experiment in which the identity of the base that templates for the 5′ nucleotide was either adenine, cytidine, guanine or thymine and the initial 5′ nucleotide was either a ribonucleotide, deoxyribonucleotide or a mixture of ribo and deoxyribonucleotides (10:1 and 1:10) (Fig. 2B, lanes 1 to 17, Fig. S9). In this experiment, AtPrimPol was unable to start a primer with a 5′-UTP or 5′-dTTP (Fig. 2B, lanes 2 to 5), whereas moderate incorporation was observed when 5′-GTP or 5′-dGTP were used as initial nucleotides (Fig. 2B, lanes 6 to 9). When 5′-GTP or 5′-dGTP are used as substrates, they are competing with the labeled ^32^P-dGTP decreasing the amount of labeled product. Even in large excess of competitor or the radioactive label, reactions incubated with AtPrimPol using 5′-GTP or 5′-dGTP generate elongated primers suggesting that 5′-GTP or 5′-dGTP can be used as initial nucleotides. 5′-dCTP or 5′-CTP were effectively used for AtPrimPol (Fig. 2B, lanes 10 to 13). Although, 5′-dCTP or 5′-CTP were used as initial nucleotides, the greater amount of primer formation was observed with ATP or dATP at the 5′ position, indicating that as HsPrimPol, AtPrimPol preferentially uses ATP or dATP as its initial nucleotide. A comparison between reactions incubated solely with ATP or dATP and labeled dGTP shows a similar amount of elongated primers but more nascent products of 2 and 3 nt are observed when using ATP (Fig. 2B, lanes 14 and 15). Our results suggest that AtPrimPol prefers ATP as initiating nucleotide over dATP because more primer products are synthesized in the presence of ATP^21,38^. Di and trinucleotides are more abundantly synthesized when ATP, but not dATP, is solely or majoritarily present in the reaction (Fig. 2B, lanes 14 and 16).

### AtPrimPol localizes at the nucleus, mitochondria and plastids

**Figure 3:**
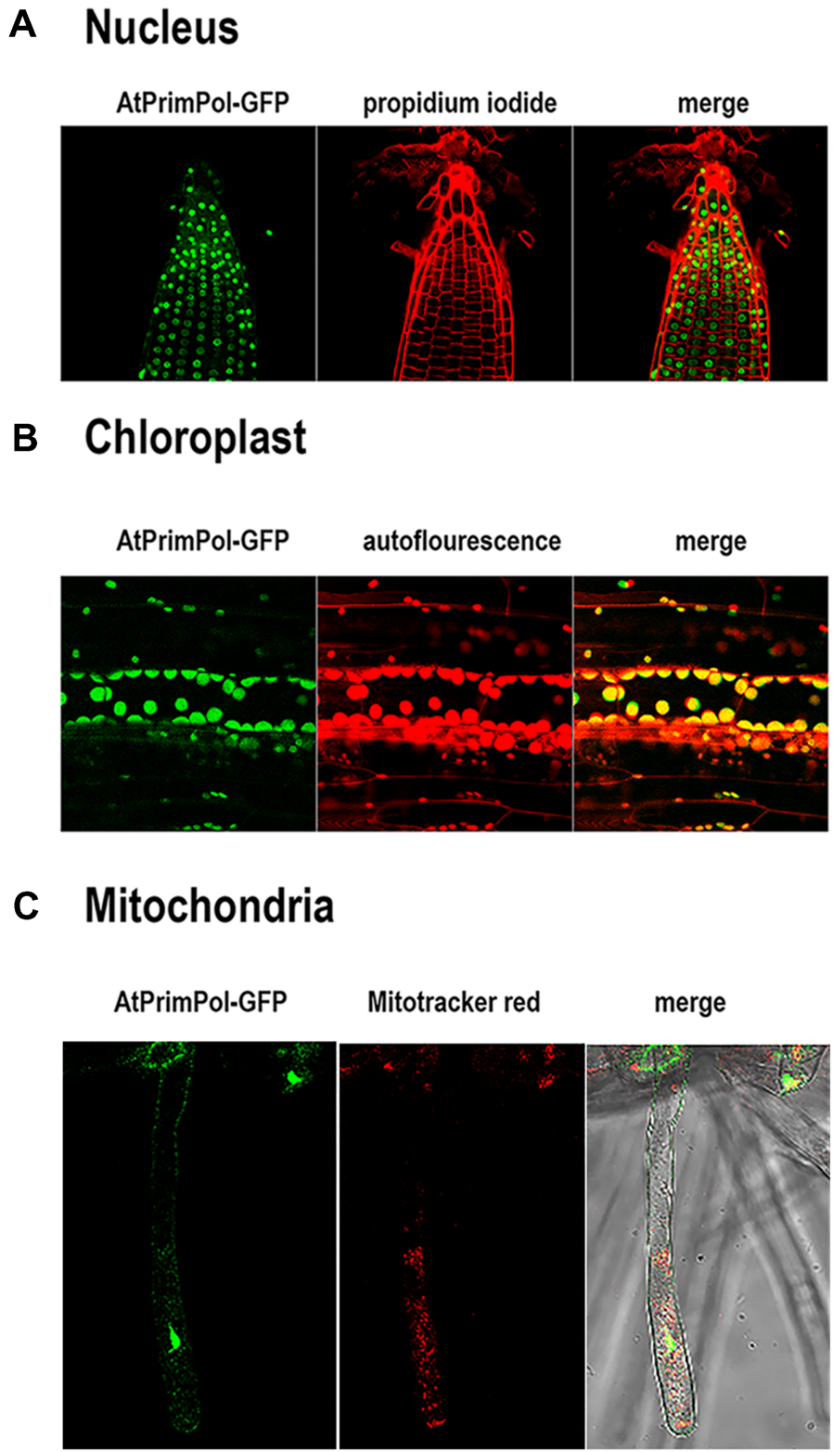
Subcellular localization of PRIMPOL-GFP. Localization of PRIMPOL-GFP in the nucleus of root cells (**A**), the chloroplasts of hypocotyl cells (**B**) and the mitochondria of root hairs (**C**).

In order to evaluate the sub-cellular localization of AtPrimPol, we constructed a protein fusion containing a GFP fused to the C-terminal of AtPrimPol under the control of 35S promoter (*35S::PRIMPOL-GFP*; Fig. 3). A close view of primary root shows that AtPrimPol-GFP localizes into the nucleus of cells at the root tip (Fig. 3A). By exploring cells at the hypocotyl of the same seedlings, we observed that AtPrimPol-GFP (Fig. 3B) colocalizes with the autofluorescence signal of chlorophyll from the chloroplasts of these cells (Fig. 3B). Finally, a close-up view of root hairs of seedlings stained with Mitotracker, shows a co-localization between AtPrimPol-GFP and mitochondria. (Fig. 3C). With the aim to corroborate the plastid and mitochondrial localization of AtPrimPol, we crossed independently plants from *35S::PRIMPOL-GFP* lines with of *35S::COX4-CFP* and *35S::NrRbcS-CFP* lines. The former is a specific marker for mitochondria, while the latter is a marker for chloroplasts^39^. F3 seedlings from such crosses were analyzed and we confirmed that AtPrimPol-GFP colocalizes with COX4-CFP at the mitochondria in both hypocotyl cells (Fig. S5A–D and insets) and root hairs (Fig. S5E–H and insets). We also observed colocalization of AtPrimPol-GFP with NrRbcS-CFP at chloroplasts in cells of the hypocotyl (Fig. S5I–L) and cotyledons (Fig. S5M–P).

### AtPrimPol efficiently bypasses DNA lesions with low fidelity

**Figure 4:**
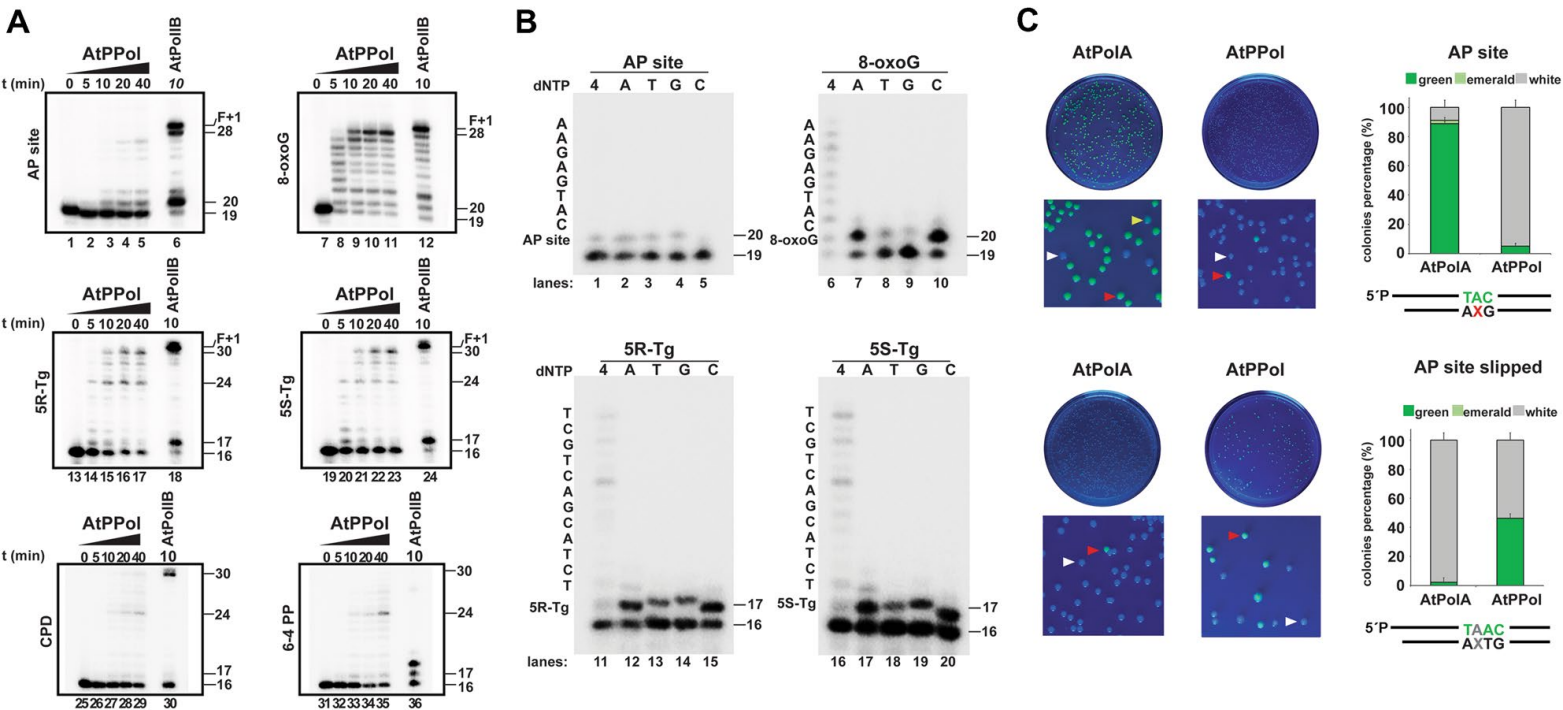
AtPrimPol is a TLS DNA polymerase, (**A**) time course reaction from 10 to 40 min showing primer extension by wild-type AtPrimPol, primer extension corresponds to 27 nt, one nucleotide shorter than full extension, whereas AtPolIB exo^−^ extends to 28 nt and one nucleotide beyond. For 8-oxoG, polymerization products were observed from 5 to 40 min. For 5*R* and 5*S* thymine glycol isomer, PrimPol full-length was capable to extend up to 30, whereas AtPolIB exo-generated 30 nts full extension and 31 nts, but also displayed nucleotide incorporation product of 17 nt. For CPD and 6–4 PP, wild-type AtPrimPol showed a weak intermediate DNA product from 10 to 20 min (**B**) A single nucleotide insertion reactions were performed using 10 nM of dsDNA substrate (AP site, 8-oxoG, 5*R*-Tg or 5*S*-Tg) mixed with 200 nM of AtPrimPol. Individual reactions were initiated by addition of individual nucleotides and stopped at 10 min. For AP site, dAMP, dTMP and dGMP were incorporated with similar preference (lane 2, 3 and 4); for 8-oxoG, dAMP and dCMP were inserted preferentially (lane 7 and 10); for 5*R*-Tg and 5*S*-Tg isomer, wild-type PrimPol inserted randomly A, T, G or C, with a slight preference by dAMP and dCMP (lane 12 to 15 and 17 to 20). (**C**) *E. coli* BL21 (*DE3*) strain overexpressing a pET19:eGFP construct in which the chromophore sequence was replaced an A(AP site)G codon-like, and replicated by AtPolIA or AtPrimPol (upper panel), in the bottom panel, the template harbors a A(AP site)TG codon-like (Fig. S6C, D). Red arrows indicate overexpressing GFP colonies, white arrows a colony overexpressing nonfunctional GFP and yellow arrows indicates an emerald colony. Graphic of the relative percentage of white, emerald, and green colonies counted by triplicate. Original data used to compose this figure is present in Fig. S10.

PrimPols from *H. sapiens* and *T. brucei* bypass DNA lesions like abasic sites (AP) and 8-oxo guanosine (8oxoG)^21,22,40^. Given the presence of reactive oxidative species and metabolic byproducts in organelles that potentially damage mitochondrial and plastid genomes, we investigated if TLS is a conserved property in AtPrimPol. We tested TLS on 8-oxoguanine (8oxoG), an abasic site (AP), and thymine glycol (Tg) using a 40-fold excess of enzyme over primer-template and AtPolIB exo^−^ as a positive control (Fig. 4A, Fig. S10). Under these experimental conditions, AtPrimPol is able to bypass an abasic site, however with limited efficiency in comparison to an undamaged template (Fig. 1C, 4A lanes 1 to 5). The length of the extension product synthetized by AtPrimPol is one nucleotide shorter that the expected full extension product of 28 nts synthesized by the control AtPolIB exonuclease deficient (exo^−^)^14^ (Fig. 4A, lanes 1 to 6). This suggests that the ability to skip an AP site is a conserved feature in PrimPols^38,41^. AtPrimPol synthesizes a full-length product at an 8oxoG template and with greater efficiency than an AP site (Fig. 4A, lanes 7 to 12) as is the case for its human counterpart^38,41^. Thymine glycol (Tg) is a strong block or replicative DNAPs^42^. Two independent groups have shown that HsPrimPol is blocked at a Tg using Mg^++^ as cofactor^22,43^. However, HsPrimPol bypasses a Tg lesion using Mn^++^ (41). In our hands, AtPrimPol is able to bypass both (5R, 6S) and (5S, 6R) Tg isomers and perform full-length primer extension (Fig. 4A, lanes 13 to 24). HsPrimPol executes TLS across a cyclobutane pyrimidine dimer (CPD) and a (6–4) pyrimidine pyrimidines (6–4 PP) by skipping the lesion^24^. To investigate if this property is conserved in AtPrimPol, we tested TLS across CPD and 6–4 PP using the same substrates previously used for HsPrimPol^24^. AtPrimPol bypassed both CPD and 6–4 PP, however the main product was 6 nts shorter than the expected full-length product of 30 nts^38^. The abilities of HsPrimPol and AtPrimPol to performs TLS at UV-generated lesion are related to primer reannealing to microhomologous template regions beyond the lesion, mediated by PrimPol^38^.

As TLS at an AP generates a product that is one nucleotide shorter than the predicted full-length product, we decided to investigate the identity of the nucleotide that is incorporated when AtPrimPol encounters an abasic site. We performed a reaction using individual nucleotides at an AP (Fig. 4B, lanes 1 to 5, Fig. S10). When we used a primer paired just before the AP site, we found that AtPrimPol incorporates dAMP, dTMP, and dGMP with similar efficiency and is not able to incorporate dCMP (Fig. 4B, lanes 1 to 5). When we repeated this experiment using another template sequence harboring the same AP site but a different template sequence (Figs. S6A,B, S14), we noticed that adenine and guanine were incorporated with the same efficacy, suggesting that AtPrimPol follows either the A-rule (inserting dA opposite the abasic site^44^) or copies the next templating base (dC), generating a -1 deletion product. Thus, AtPrimPol may use template bases in a primer realignment mode to direct primer templated synthesis or direct synthesis according to the context of the template sequence^38^. As previously demonstrated by Martínez-Jiménez and collaborators HsPrimPol bypasses AP sites by using the next available template as a substrate^38^. In contrast to the non-instructive character of an AP site, AtPrimPol preferentially incorporates dAMP or dCTP opposite an 8oxoG (Figs. 4B, lanes 6 to 10, S10). AtPrimPol incorporates all dNTPs opposite a Tg lesion. A detailed observation of TLS abilities at a Tg lesion show the presence of a 24 nts product and a full-length product of 30 nts. The appearance of the 24nts product is related to primer realignment in which different bases direct the character of the incoming nucleotide (Fig. 4B, lanes 11 to 20, Fig. S10).

The fact that AtPrimPol incorporates dNTPs with similar efficiency at an abasic site and that the length of the product is one nucleotide shorter than the full-length product on a canonical or an 8oxoG template, prompted us to investigate the relative efficiencies by which AtPrimPol skips the abasic site or uses the next template base. To investigate this question, we took advantage that more than 95% of an AP in *E. coli* is repaired before it can be used as a template^45,46^. In this experiment, we used a primer hybridized to a template harboring a modified coding sequence for eGFP. Primer extension was executed on a template containing an ATG codon that codes for the Tyr chromophore or in a template in which this codon is replaced by an A(AP)G-like codon. If a DNA polymerase incorporates a dAMP, accordingly to the “A-rule”, opposite to the AP site, the A(AP)G-like codon would code for Tyr and fluorescence will be established. In a parallel experiment, we used a primer hybridized to a templated harboring A(AP)TG-like codon. If a dAMP or other base is inserted opposite the AP site, the chromophore will not be assembled, and white colonies would be observed. However, if the AP site is skipped and a dAMP is incorporated across the template, the ATG codon would be restored, and green colonies would be observed (Fig. S6C,D). An A(AP)G containing oligonucleotides amplified by AtPolIA exo^−^ yield 90% of green colonies (355 out of 394) and 2% of emerald colonies (9 out of 394), in contrast oligonucleotides extended by AtPrimPol only yield 5% of green colonies (14 out of 295) (Fig. 4C). An experiment using an A(AP)TG-like codon with containing oligonucleotides amplified with AtPolIA exo^−^ results in only 2% of green colonies, whereas its amplification by AtPrimPol resulted in 62% of green colonies. Overall the data indicates that AtPolIA exo^−^ preferentially inserts an Ade opposite and abasic site and has little propensity to skip this lesion, on the other hand AtPrimPol preferentially skips an abasic site and use the next available base (T) as a template. The observed 38% of white colonies may correspond to the repaired A(AP)TG-like codon that is converted to an ATTC-like codon that shifts the open reading frame of eGFP (Fig. 4C). A control experiment in which the A(AP)G templated, is annealed with an oligonucleotide harboring the TAC sequence results in the appearance of 99% of green colonies, indicating that the AP is readily repaired and that AP site is substituted by a T (Fig. S6E) and as expected a control experiment in with the ATG codon is replaced by ATT-codon (Ile) annealed to a “TAA” yields only white colonies. An experiment in with the ATT-codon (Ile) and an oligonucleotide harboring the TAC sequence are annealed produces 49% and 51% of green colonies indicating that both DNA strands are independently replicated. This data resembles the 60% of polymerization errors observed in a β-galactosidase derived system using a single-stranded M13 template^47^. To investigate the fidelity during nucleotide incorporation by AtPrimPol we used four primer templates in which the identity of the next template bases changes using Mg^++^ or Mn^++^ as cofactors. AtPrimPol preferentially incorporates dNMPs following the Watson–Crick rules, however, AtPrimPol also misincorporates specially in the presence of Mn^++^ (Figs. S7, S15).

### AtPrimPol executes MMEJ with limited strand-displacement activities

**Figure 5:**
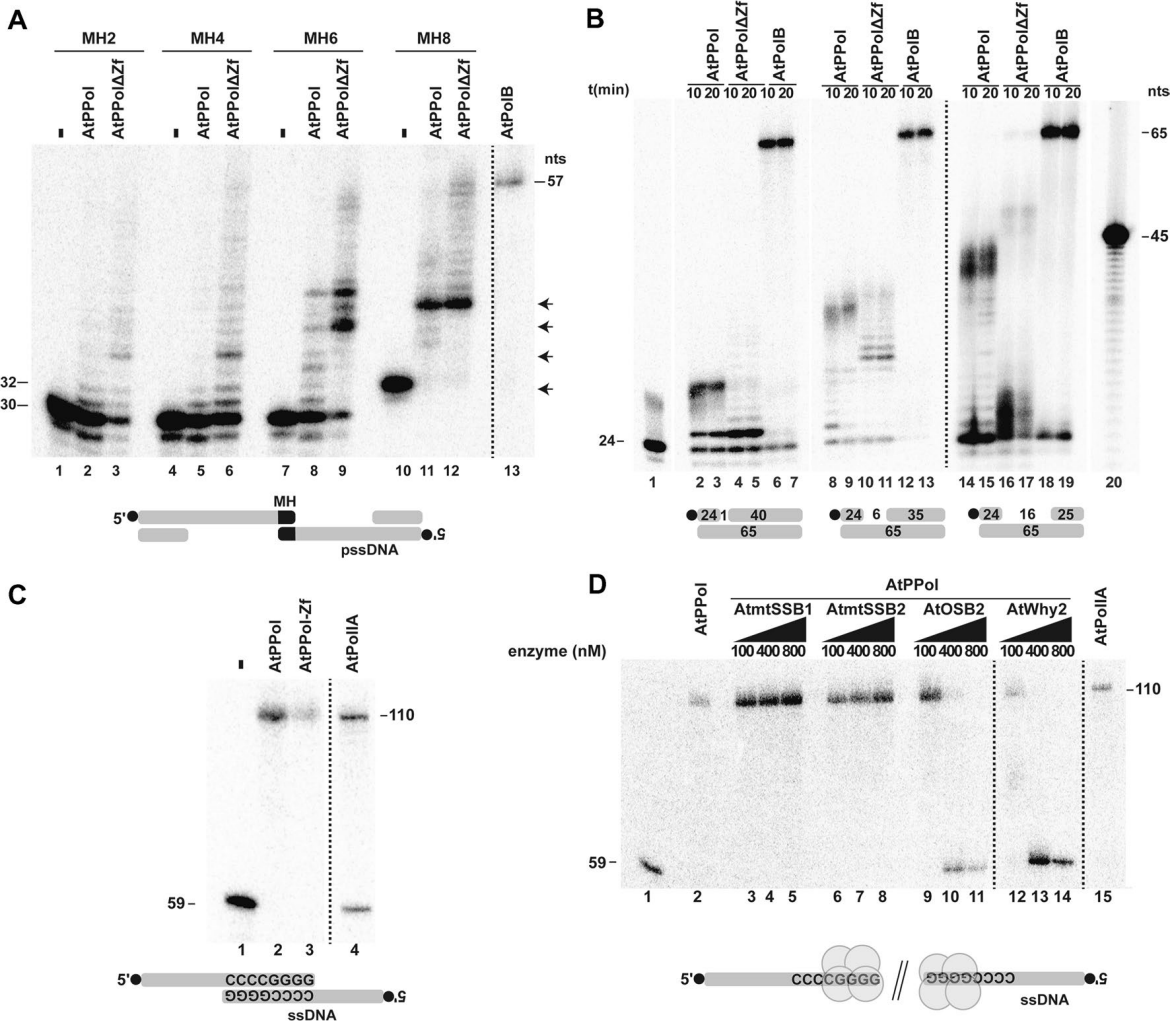
AtPrimPol performs microhomology-mediated end-joining and moderated strand-displacement. Reactions were incubated at 10 (Strand displacement assay), 20 (strand displacement assay) 30 min (microhomology assay). All reactions were loaded onto 17% polyacrylamide denaturing gel and analyzed by phosphorimaging. (**A**) AtPrimPol at a concentration of 400 nM was able to generate intermediate products (lanes 2, 5, 8, and 11) into the gap created during synapse formation between pssDNAs with different length of microhomology sequence (2, 4, 6 and 8 nts), in contrast with the deficient Zn^++^ finger mutant of AtPrimPol, AtPPolΔZF, that improved the primer extension at the same concentration (lanes 3, 6, 9, and 12). As a control of microhomology, reaction containing 100 nM of AtPolIB was capable to fill the gap and exhibited strand-displacement activity, showing a final product up to 57 nts (Lane 13). (**B**) Strand-displacement on gapped substrates, AtPrimPol fills a 1-nt gap but is unable to displace the blocking oligonucleotide. In contrast, AtPPolΔZf fills the 1-nt gap and displaces the blocking oligonucleotide with minimal efficiency (lanes 2 to 5). A similar pattern is observed on substrates with a 6-nt gap (lanes 8 to 11) or a 16-nt gap (lanes 14 to 17). An exonuclease deficient version of AtPolIB was used as a positive control for strand displacement (Lanes 6, 7, 12,13, 18, and 19). (**C**) MMEJ activities on, ssDNA. Both AtPrimPol (Lane 2) and of AtPPolΔZF (Lane 3) execute MMEJ on 3′ overhang from ssDNA with 8 nts as microhomology sequence (5′-CCCCGGGG-3′) and produce the 57 nts product as AtPolIB. (**D**) In presence of an increasing concentration of single stranded DNA binding proteins from *A. thaliana,* the microhomology reaction using ssDNA as substrate was not affected by AtmtSSB1 or AtmtSSB2 and severely hampered by AtOSB2 and AtWhy2. Original data used to compose this figure is present in Fig. S11.

In order to investigate a possible role of AtPrimPol in MMEJ we assayed primer-templates resembling partially resected DSBs (pssDNA)^48^. These substrates contain microhomologous regions of 2, 4, 6 and 8 base pairs. These substrates have annealing temperatures higher than 37 °C and if annealed they can synthesize products of 54 and 58 nts^15,48^. AtPrimPol and the AtPPol∆Zf mutant are able to use a pssDNA with 2 nts of microhomology as a template (Fig. 5A, Fig. S11). As is the case for other DNA polymerases, AtPrimPol uses pssDNA substrates more efficiently as the length of their microhomologous region increases (Fig. 5A, Fig. S11)^15,48^. AtPrimPol incorporate few nucleotides at the double-stranded junction of the annealed pssDNA, indicating that the strand-displacement properties of this enzyme are insufficient to completely remove the annealed non-template strand (Fig. 5A, Fig. S11). This phenomenon is better observed on templates with 6 and 8 nts of microhomology as extension products of few nucleotides beyond the microhomologus region are synthesized specially in reactions incubated with the AtPPol∆Zf mutant (Fig. 5A, lanes 8, 9, 11, 12).

To further corroborate the moderate (or minor) strand-displacement activities of AtPrimPol and the AtPPol∆Zf deletion mutant, we used a primer-template arrangement in which the primer strand and the blocking oligonucleotide are separated by 1, 6, and 16 nts and assayed extension reactions side by side using AtPolIB exo-, a DNA polymerase that exhibits strong strand-displacement (Fig. 5B, Fig. S11)^17^. AtPrimPol and the AtPPol∆Zf deletion mutant fill the gap of the 1-nt gapped substrate before the blocking oligonucleotide. AtPrimPol is unable to displace the blocking oligonucleotide, whereas AtPPol∆Zf displaces the lesion albeit with limited efficiency (Fig. 5B, lanes 2 to 5). This limited efficiency contrast with the strong strand-displacement exhibited by AtPolIB exo−, that efficiently synthesized a full-length product after displacing the blocking nucleotide (Fig. 5B, lanes 6 and 7). This pattern is maintained on gapped substrates with gaps of 6-nts and 16-nts (Fig. 5B, lanes 8 to 19), corroborating the limited efficiency of full-length AtPrimPol in displacing a blocking oligonucleotide and that the removal of the Zn^++^ finger increases the strand-displacement abilities of this enzyme. The deletion of the Zn^++^ finger subdomain in AtPrimPol promotes a limited or minor strand-displacement activity, as this modified enzyme is able to add few nucleotides on templates with microhomologous sequences and templates that have an oligonucleotide directly blocking the advance of the polymerase (Fig. 5A,B). We attributed that the added nucleotides are consequence of limited strand-displacement and not to enhanced DNA polymerization activity, as the AtPrimPol and AtPPol∆Zf present similar nucleotide incorporation activities on a primer-template without a blocking oligonucleotide (Fig. 1C).

### AtPrimPol executes MMEJ preferentially on single-stranded templates

As AtPrimPol is deficient in strand-displacement, we asked if AtPrimPol would use a single-stranded template with a microhomologous region as a substrate. Both AtPrimPol and the AtPPol∆Zf are able to use a single-stranded substrate with a microhomologous region of 8-nts (Fig. 5C, Fig. S11). HsPrimPol is recruited to nuclear and mitochondrial DNA by RPA and mtSSB, respectively, albeit is not clear how human mtSSB interact with HsPrimPol (23, 30). Besides two canonical mtSSBs, plant mitochondria harbor two different families of single-stranded binding proteins enzymes dubbed Organellar Single-Stranded Binding protein (OSB) and Whirlies^49–53^. In order to study a possible interaction between AtPrimPol and plant SSBs, we investigated if plant mitochondrial SSBs (AtmtSSB1 and AtmtSSB2), plant mitochondrial OSB2 (AtOSB2) and Whirly 2 (AtWhy2) physically interact with AtPrimPol by thermophoresis. All mtSSBs at a concentration of 2 μM were unable to interact with AtPrimPol (data not shown). Under our experimental conditions AtPrimPol converts 100% of the MMEJ-annealed template into a full-length product of 110 nts. As mtSSBs and AtPrimPol do not interact, mtSSBs may modulate MMEJ by preventing the access of AtPrimPol to single-stranded DNA. The addition of AtmtSSB1 and AtmtSSB2 does not have an effect on the MMEJ abilities of AtPrimPol. In contrast, both AtOSB2 and AtWhy2 at concentrations greater than 100 nM impinge MMEJ (Fig. 5D, Fig. S11). This inhibition can be related to higher binding affinity of AtOSB2 and AtWhy2 to ssDNA in comparison to AtmtSSB1 and AtmtSSB2^15^. The regulation of AtPrimPol supports a role of mitochondrial SSBs in reducing genomic rearrangements in plant organellar DNA^35,54^.

### AtPrimPol generate primers that are elongated by plant organellar DNA polymerases

**Figure 6:**
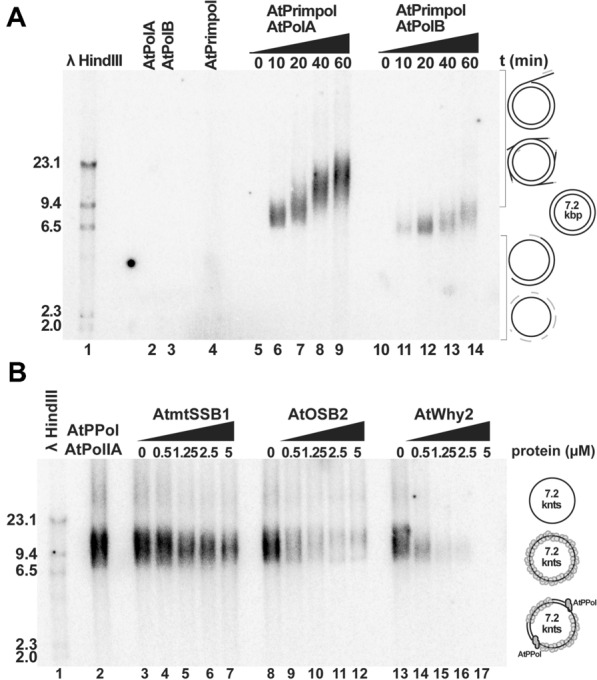
AtPrimPol synthetizes primers that are elongated by plant organellar DNA polymerases. (**A**) A coupled primase-DNA polymerase reaction containing 200 nM of AtPrimPol and 200 nM of AtPolIA or AtPolIB was setup. The reactions were incubated the presence of dNTPs and labeled with dGTP, using as template a ssDNA M13 vector, reactions were loaded in a 0.6% agarose gel. Both coupled reactions AtPrimPol-AtPolIA (lanes 5–9) and AtPrimPol-AtPoIlB (lanes 10–14) were able to replicate the ssDNA vector of 7.2 kbp at the lowest reaction time. Increasing the reaction time leads to an increase of size of the DNA product (up to 23 kbp) due to the strand-displacement activity of the POPs. AtPrimPol (lane 2), AtPolIA (lane 3) and AtPolIB (lane 3) on their own, were unable to synthetize the full-length DNA product at the highest reaction time (lanes 2–4). (**B**) M13 ssDNA was incubated with increasing concentrations (0, 620, 1250, 2500 y 5000 nM) of organellar single-stranded binding proteins (OSSBs) AtmtSSB1, AtOSB2 and AtWhy2 at room temperature during 30 min, afterwards a mixture of 200 nM of AtPrimPol and AtPoIlA was added and the reaction was incubated at 37 °C during 40 min. Reactions were loaded onto a 0.6% agarose gel. The reactions in presence of AtWhy2 (lanes 13–17), showed that this protein efficiently blocks or inhibit the replication from 620 nM where the half of product was reduced. In the AtOSB2′ s reactions (lanes 8–12) the fall of de product is more slowly, even at the highest concentration some product was produced. Whereas the reactions with AtmtSSB1 (lanes 3–7) showed that AtPrimPol-AtPolI were able to replicate the ssDNA vector while the concentration of AtmtSSB1 was increasing. Original data used to compose this figure is present in Fig. S12.

We were curious to test if AtPrimPol could synthesize DNA primers for plant organellar DNA polymerases, AtPolIA and AtPolIB—The latter as an alternative to the synthesis RNA primers by the plant organellar primase-helicase, AtTwinkle, during the assembly of a T7-like replisome in plant organelles^16,55^. Aiming to investigate if AtPrimPol synthesizes primers that are elongated by AtPolIs, we used a single-stranded circular M13 in the presence of fixed concentrations of AtPrimPol and AtPolIs (Fig. 6A, Fig. S12). AtPrimPol, AtPolIA and AtPolIB alone are unable to synthesize a large polymerization product (Fig. 6A, lanes 2, 3, 4). However, AtPrimPol in combination with either AtPolIA (Fig. 6A, lanes 6 to 9) or AtPolIB (Fig. 6A, lanes 11 to 14) synthesize an initial product of approximately 7.2 kbp. In the case of AtPolIA, this initial product reaches nearly 20 kbs and in the case of AtPolIB it reaches 10 kbs. These phenomena correlate with the preferred extension of primers generated by AtTwinkle by AtPolIA in comparison to AtPolIB^14^ and the proposed role of AtPolIA in DNA replication and AtPolIB in DNA repair^56^. The primer activity of AtPrimPol has the potential to create new origins of replication, transversions and deletions. Thus, its activity has to be regulated. Plant organellar single-stranded DNA binding proteins, mtSSBs, Whirlies, and OSB decrease the formation of rearrangements at microhomologous sequences and short repeats^56^ AtPrimPol activity may be regulated by blocking its access to single-stranded DNA and alternatively, plant organellar single-stranded DNA binding proteins may block primer extension by AtPolIs. Here we measured the formation of elongation products on a single-stranded circular M13 by AtPrimPol and AtPolIA in the presence of increasing concentrations of AtmtSSB1, AtOSB2, and AtWhy2 using protein concentrations that rank from 0.5 to 5 μM (Fig. 6B, Fig. S12). Assuming that all proteins bind to a segment of 10-pb of single-stranded DNA, a concentration of 0.72 μM of these proteins would be sufficient to fully coat the single-stranded M13 template. All single-stranded binding protein AtmtSSB1, AtOSB2, and AtWhy2 impinge the synthesis of long DNA products by the combined action of AtPrimPol and AtPolIA. This blockage is specially observed in AtOSB2 and AtWhy2 that bind ssDNA with a nanomolar affinity. The residual DNA extension activity observed in reactions incubated by AtmtSSB1 could be due to its weaker binding to single-stranded DNA with respect to AtOSB2 and AtWhy2^15^.

## Discussion

Photosynthetic organisms, like algae and flowering plants, are constantly exposed to the sun and therefore to UV irradiation. Plant harbor TLS DNAPs like DNA polymerase η and DNAP ζ that cope with UV-mediated DNA damage, especially in stem cells^8,57,58^. PrimPol is the most recent DNA polymerase identified in metazoans. Its evolutionary relationships with the AEP superfamily of primases agrees with its unique role in repriming stalled replication forks by synthesizing “de novo” DNA primers^21,21,28–32,59^. According to the postulate “*PrimPol is an ancient eukaryotic gene*”^21^, herein we show that his enzyme was present in eukaryotes before the divergence between plants and animals. HsPrimPol is important in maintaining replication forks in the nucleus and avoiding sensitivity to DNA lesions like UV light. Its role in the nucleus is specially visible in the absence of DNA polymerase η^60^. Metazoan PrimPols harbor both nuclear and mitochondrial localization and PrimPols in plants have added a targeting peptide for mitochondrial and chloroplastic localization (Fig. 1, Fig. S2). Plant and metazoan PrimPols share a high degree of similitude in their APE and Zn++ finger subdomains and their main differences in primary structure are located at the C-terminal RPA binding region. In humans RPA is a heterotrimer composed of the subunits dubbed RPA70, RPA32 and RPA14^61^. Human PrimPol binds to RPA70 via two binding motifs dubbed RPA-binding motifs A and B, in which RPA-binding motif A plays a predominantly role using hydrophobic and negatively charged amino acids^35^. Plant PrimPols shows conservation in the RPA-binding motif A, but not the RPA-binding motif B (Fig. S3). The lack of conservation between RPA binding motifs may be related to numerous events of gene duplication of RPA70 in *Arabidopsis* that contains five RPA70 paralogs^62^. The duplication events of RPA32 and RPA14 could produce up to 20 RPA heterotrimers in Arabidopsis^63^.

AtPrimPol as a DNA polymerase bypasses strong-blocking lesions like AP site, CPD, and 6–4 PP by skipping the lesion. Using a GFP reporter we show that AtPrimPol almost exclusive skips an abasic site using the next templated (or templating) base. (Fig. 4A). The proposed MMEJ abilities of AtPrimPol were corroborated using pssDNA substrates (Fig. 5). As DNA polymerases θ and λ, AtPrimPol is able to use a pssDNA containing only 2 nts of homology as substrates. This reaction is more effective in polymerases lacking the Zn^++^ finger domain, suggesting the possibility that alternative splicing may regulate MMEJ in vivo, as two of the three putative AtPrimPol isoforms lack its Zn^++^ finger. Although AtPrimPol is able to use pssDNA substrates, this enzyme is not able to displace a blocking oligonucleotide, but could be stimulated by other accessory proteins like in HsPrimPol. Arabidopsis plants lacking a functional AtPolIB in combination with insertional lines in the genes encoding for AtWhy1 and AtWhy3 increase the formation of MMEJ re arrangements in chloroplast^56^. Interestingly this phenomenon is not observed in insertional lines of AtPolIA. The reduced fidelity during nucleotide incorporation of AtPolIB in comparison to AtPolIA, suggests a predominant role of AtPolIB in DNA repair rather than in replication^13^. As AtPolIA and AtPolIB have similar efficiency for MMEJ^15^, it is plausible that in plant organelles in the absence of AtPolIB, AtPrimPol is overexpressed leading the establishment of MMEJ that are further elongated by AtPolIA.

Human PrimPol is primordial to promote mitochondrial DNA replication, even in the absence of DNA damage^59,60,64^. Plant mitochondrial genomes suffer extensive and rapid rearrangements that could lead to the evolution of new genes in a phenomenon often associated with cytoplasmic male sterility (CMS)^65–69^. These genes are primarily originated by homologous recombination; however, it is clear that MMEJ and non-homologous end-joining (NHEJ) play a role in their formation^70,71^. Given that AtPrimPol can use single and pssDNA substrates with limited microhomology and its mitochondrial localization, we were curious to test if mitochondrial single-stranded binding proteins (mtSSBs) specific to plant mitochondria could modulate its MMEJ activity. In contrast to metazoan mitochondria, plant organellar harbor three different families of mtSSBs: (1) a duplicated copy of canonical SSBs (AtmtSSB1 and AtmtSSB2, (2) four paralogs of a protein dubbed Organellar Single-Stranded Binding protein (OSB) and (3) three paralogs of a protein dubbed Whirly^50,51,72^. We tested the effect AtmtSSB1, AtmtSSB2, AtOSB2) and AtWhy2, as these proteins are located in mitochondria, in regulating MMEJ promoted by AtPrimPol. Both OSB2 and Why2 hamper MMEJ by AtPrimPol, a phenomenon that correlates with the role of these proteins in avoiding re-arrangements at microhomologous regions^49,52,56^. The ability AtOSB2 and AtWhy2 in avoiding primer extension mediated by AtPrimPol also correlates with the role of these proteins in avoiding DNA re-arrangements in plant organelles and the ability of AtPrimPol to synthesize primers that are elongated by organellar DNA polymerases made it suitable in avoiding replication fork collapse.

In sum, our data suggests that AtPrimPol plays a predominant role in securing correct DNA replication in plant genomes, especially in organelles that lack nucleotide excision repair pathway necessary to remove bulky adducts like CPD of 6–4 PP.

## Materials and methods

### Cloning and purification

The coding region of AtPrimPol was synthetically optimized for its expression in bacteria (Biomatik). The coding region corresponding to residues 87 to 614 was PCR amplified and subcloned between *Nde* I and *Bam* HI restriction sites of a modified pET19b vector. Codon-optimized AtPrimPol was recombinantly induced expressed at 14 °C in an *Escherichia coli* BL-21 (*DE3*) strain supplemented with a pKJE7 plasmid. For protein purification, one liter of cell culture was harvested and resuspended in 30 ml of lysis buffer (50 mM Tris–HCl pH 8.0, 150 mM NaCl, 10% glycerol, 5 mM imidazole and 1 mM PMSF) supplemented with 0.5 mg/ml lysozyme, and incubated at 4 °C for 30 min. Cells were lysed using two cycles of freeze–thawing. The lysate was centrifuged at 12,000 rpm for 45 min at 4 °C. The cell-free extract was purified by immobilized metal affinity chromatography (IMAC) using a 1 ml pre-packed column. The clarified lysate was loaded onto the IMAC column and the column was extensively washed with 50 ml of lysis buffer using an imidazole gradient (from 10 to 150 mM). AtPrimPol was eluted with 3 ml of lysis buffer supplemented with 500 mM imidazole and dialyzed in 50 mM Tris–HCl pH 8, 50 mM NaCl, 1 mM DTT, 10% glycerol and 1 mM PMSF. The dialyzed protein was loaded onto a heparin column equilibrated with dialysis buffer and washed with 100 volumes of a NaCl gradient (from 20 to 300 mM). AtPrimPol was eluted with 2 ml of dialysis buffer supplemented with 1 M NaCl. The eluted protein was dialyzed in 50 mM Tris HCl pH 8.0, 500 mM NaCl, 1 mM DTT, 1 mM PMSF, and 50% glycerol and stored at − 20 °C. The AtPrimPol ΔZnF mutant, that corresponds to residues 87 to 387 as this deletion eliminates the linker region, Zn++ finger and RPA binding domain, was subcloned using BamHI and NdeI restriction sites into the pCri-1b vector. AtPrimPol ΔZnF mutant was expressed and purified as the wild-type AtPrimPol.

### DNA and RNA synthesis

Reactions mixtures were incubated primer-templates annealed oligonucleotides with AtPrimPol in a reaction buffer containing 150 μM NTPs, 250 μM dATP, 250 μM dTTP, 250 μM dCTP, 10 μM dGTP, and 2 μCi [α-32P]-dGTP (3000 Ci/mmol). Templates were present at 2.5 nM using varying AtPrimPol concentrations (indicated in each figure legend). Reactions were carried out at 37 °C for 30 min and analyzed on denaturing polyacrylamide gels.

### Translesional DNA synthesis

For AP site and 8-oxoG, a 19-mer radiolabeled primer 5′ TGT TAG CAG ACA AGC CGA T 3′ was annealed with 28-mer template DNA 5′ AAG AGT AC **X** ATC GGC TTG TCT GCT AAC A 3′ containing the lesion denoted by an X. For thymine glycol isomers, a 16-mer radiolabeled primer 5′-CAC TGA CTG TAT GAT G-3′ was annealed with a 30-mer 5′-CTC GTC AGC ATC T**X**C ATC ATA CAG TCA GTG-3′, where **X** indicate 5*R*-Tg or 5*S*-Tg, and for CPD or 6–4 PP photoproducts the same 16-mer radiolabeled primer was annealed with a 30-mer 5′-CTC GTC AGC ATC **XX**C ATC ATA CAG TCA GTG-3′. TLS reactions contained 10 nM of dsDNA substrate were mixed with 400 nM of AtPrimPol or 20 nM of AtPolIB exo^−^ and pre-incubated for 5 min at 37 °C. Reactions were initiated by addition of 200 μM dNTPs and stopped at 5, 10, 20 and 40 min and for AtPolIB exo^−^ was a single time of 10 min. Reaction mixtures were run on a 17% denaturing polyacrylamide gel and visualized by phosphor imagery.

### Fidelity reactions

First, we assayed nucleotide fidelity incorporation using four undamaged dsDNA template, where the base A, T, G or C in the template is immediately after 3-OH end primer. A typical nucleotide insertion reaction was initiated by addition of 100 μM of dATP, dGTP, dTTP or dCTP in presence of 5 mM MgCl_2_ and stopped at 10 min by the addition of an equal amount of stop buffer. The same assay was performed in presence of 1 mM MnCl_2_. Single nucleotide insertion reactions were also performed using 10 nM of dsDNA substrate (AP site, 8-oxoG, 5*R*-Tg or 5*S*-Tg) mixed with 200 nM of PrimPol full-length in a buffer containing 10 mM Bis–Tris-Propane-HCl pH 7, 10 mM MgCl_2_, 1 mM DTT, 1 mM MnCl2 and pre-incubated for 5 min at 37 °C. Individual reactions were initiated by addition of 100 μM of dATP, dGTP, dTTP or dCTP and stopped at 10 min.

### In vivo GFP approach fidelity assay

To test the nucleotide incorporation opposite an AP site, we carried out a typical polymerization assay, mixing AtPolIA exo^−^ or AtPrimPol with a DNA substrate constructed with a 38-mer primer annealed to a 69-mer template harboring a modified coding sequence for eGFP. The oligonucleotide template containing an ATG codon that codes for the Tyr chromophore or another template in which this codon is replaced by an A(AP)G-like codon. To test if AP site is skipped by AtPrimPol, we used a 38-mer template annealed to a 70-mer where we put a T beside AP site resulting the A(AP)TG-like codon. Reactions were stopped by heating at 95 °C for 5 min. Products purified were digested with *Spe* I and *Kpn* I and each one of them were individually ligated into the modified plasmid pET19b harboring an eGFP sequence previously digested by the same enzymes to removing the chromophore zone. All constructions were transformed into the *E. coli* strain BL21 GOLD (DE3) and plated in LB media containing 100 μg/ml of ampicillin and incubated at 37 °C for 20 h and stored at 4 °C for 4 days to observe the green color in colonies overexpressing eGFP protein. The percentage of green, emerald or white colonies was determined. Sequencing of individual colonies was used to determine the nucleotide inserted opposite the AP site, or if the AP site was omitted.

### Microhomology and strand-displacement assays

All reactions were performed in Tris–HCl pH 7.4, 10 mM NaCl, 1.5 mM DTT, 0.1 mg/ml BSA, 10% glycerol, and 10 nM of 5 radiolabeled substrate. Enzymes were preincubated by 10 min at 37 °C and the reactions was initiated using 200 μM of dNTPs and 10 mM of MgCl_2_ and 1 mM MnCl_2._ The reactions were stopped using a stop buffer (95% formamide, 10 mM EDTA, 0.1% bromophenol blue and 0.1% xylene cyanol). All reactions were loaded onto a 17% polyacrylamide denaturing gel and analyzed by phosphorimaging.

### Coupled AtPrimPol-AtPolI reactions

AtPolIA, AtPolIB and plant organellar single-stranded binding proteins were recombinantly expressed and purified to homogeneity as previously reported^14,15^.

A coupled primase-DNA polymerase reaction containing 200 nM of AtPrimPol and 200 nM of AtPolIA or AtPolIB was performed as follows: the reactions were incubated at 37 °C during 0, 10, 20 40 and 60 min in the presence of dNTPs and labeled with 2 μCi [α-32P]-dGTP (3000 Ci/mmol) using as template a ssDNA M13 (1 nm) vector, reactions were stopped with EDTA and loaded in a 0.6% agarose gel. M13 ssDNA was incubated with increasing concentrations (0, 620, 1250, 2500 y 5000 nM) of organellar single-stranded binding proteins AtmtSSB1, AtOSB2 and AtWhy2 at RT during 30 min, afterwards a mixture of 200 nM of AtPrimPol and AtPolIA was added and the reaction was incubated at 37 °C for 40 min. Reactions were stopped with EDTA and loaded in a 0.6% agarose gel.

### Generation of organelle marker lines

Mitochondrial and chloroplast markers lines plasmids used in this study were obtained from the *Arabidopsis* stocks center (http://www.arabidopsis.org) and described in an article published previously^39^. For the co-localization analysis, transgenic Arabidopsis plants were generated using 3 week-old plants with 35S::AtPrimPol-GFP construct and transformed with plasmids based on a by the floral dipping method. Transgenic plants were screened on MS medium and glufosinate ammonium, the survived plants were transferred to soil in a greenhouse to harvest seeds. Homozygous lines were isolated at T3 generation. Fluorescence signals and images were taken using a confocal laser scanning microscope.

### Statement on the use of plants

All methods and materials regarding the use of plants were carried out in accordance with relevant international and institutional guidelines and regulations. This work complies with The International Union for Conservation of Nature (IUCN) Policy Statement on Research Involving Species at Risk of Extinction and the Convention on the Trade in Endangered Species of Wild Fauna and Flora.

## Supplementary Information

Supplementary Information.
