# Supplementary material for: Arabidopsis thaliana PrimPol is a primase and lesion bypass DNA polymerase with the biochemical characteristics to cope with DNA damage in the nucleus, mitochondria, and chloroplast
Source: Sci Rep. 2021 Oct 18;11:20582. doi: 10.1038/s41598-021-00151-7 (PMC8523556; doi:10.1038/s41598-021-00151-7)
Supplement: Supplementary file 1 — Supplementary Information. [file 41598_2021_151_MOESM1_ESM.pdf]

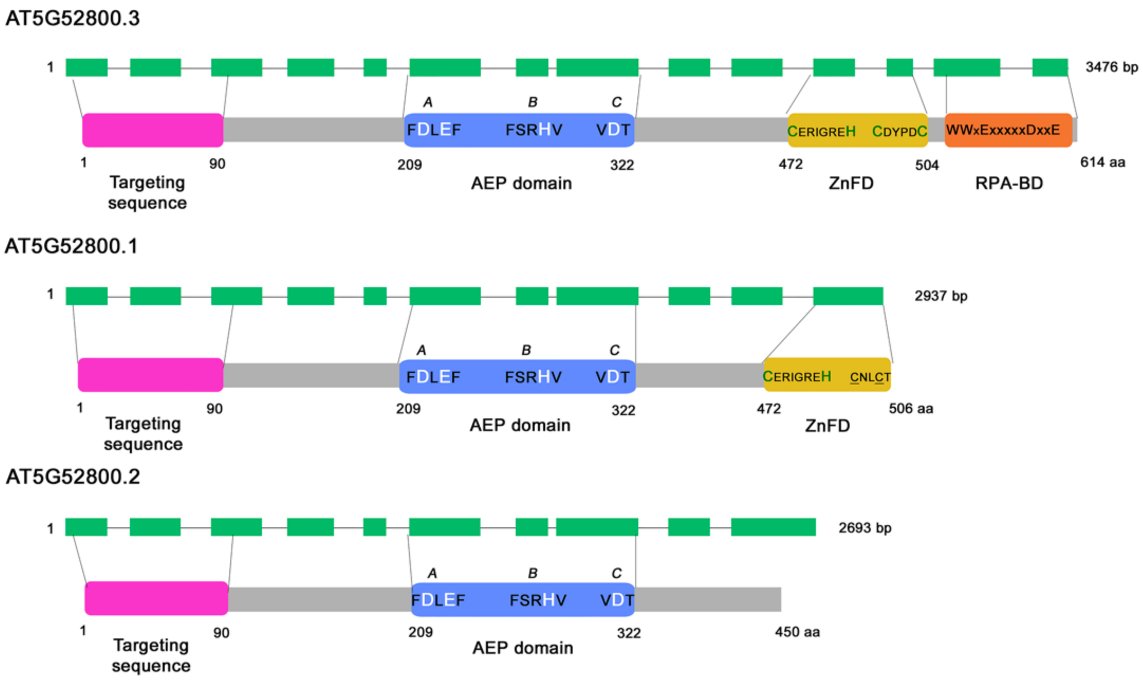

Fig. S1 Domain organization of AtPrimPol isoforms. The conserved motifs in the AEP and Zn^{++} finger subdomains are indicated. The N-terminal 90 amino acids present in AtPrimPol that contain a putative organellar targeting sequence is colored in purple.

```
             [                          SIGNAL PEPTIDE
HsaPrimpol   ------------------------------------------------------------
AthPrimpol   MSKKLSSNSMDDVDRLFQCFKCGISPPASAMREKKRSKKMLNSEYDSPVTCKKLQLSSLS 60

                                            ][AEP DOMAIN
HsaPrimpol   ------------------------------MNRKWEAKLKQIEERASHYERKPLSSVYRP 30
AthPrimpol   RLSGKRGERNQIDGSVSVDSVSGSRDVKKSVGRQFSPIVF-YGSPNGVPPKKPL-SLLRL 118


HsaPrimpol   RLS-KPEEPPSI--------WRLFHRQAQAFNFVKSCKEDVHVFALECKVGD-GQRIYLV 80
AthPrimpol   LREIRIDLSEERKAISRKGVWATFPRQEEAIKFEKRH-DNVRIFSYQDHFS--GQRRFLV 175

                                        ____MOTIF A____
HsaPrimpol   TTYAEFWFYYKSR-KNLLHCYEVIPENAVCKLYFDLEFNKPANPGADGKKMVALLIEYVC 139
AthPrimpol   SSYEEFWKRYLSMDPRHRHHYEVIQEGLPCHMYFDLEFNQKENEGKNVDEMVDILISVIL 235

                          ____MOTIF B____
HsaPrimpol   KALQELYGVNCSAEDVLNLDSSTDEKFSRHLIFQLHDVAFKDNIHVGNFLRKILQPALDL 199
AthPrimpol   EALREKYAIEGQEDWIVELDSSTKDKFSRHVIVIIPKVAFKDNSHVGAFVGELCSRIVNA 295

HsaPrimpol   LGSEDDDSAPETTGHGFPHFSEAPARQGFSFNKMFTEKATEESWTSNSKKLERLGSAEQS 259
AthPrimpol   KE-----------------------------------------------TDERLRKL--- 305

                          ____MOTIF C____
HsaPrimpol   SPDLSFLVVKNNMGEKHLFVDLGVYTRNRNFRLYKSSKIGKRVALEVTEDNKFFPIQSKD 319
AthPrimpol   -----FVHKEANDSASLLFVDTAVYSRNRCFRLALSSKAGKTSVLLPTGRFKCKDMG--- 357

                                        ]____________INTERDOMAIN REGION__
HsaPrimpol   VSDEYQYFLSSLVSNVRFSDTLRILTCEPSQNKQKGVGY-----------------F--N 360
AthPrimpol   ---ERDVFMTSLICNVES-DCEKLLVCKMESDCMKTLCFDTEVNSNNLVRDQNAQKFQLN 413

             ___________                                             ____
HsaPrimpol   SIGTSVETIEGFQCSPYPEVDHFVLSLVNKDGIKGGIRRWNYFFPEELLVYDICKYRWCE 420
AthPrimpol   ASTSDMSTSYFGGKSPFPQLDQFIESTASTGNVPGKIRCWYWFSEDGLIVYSMLRNRYCE 473

             ________ZINC FINGER DOMAIN_______
HsaPrimpol   NIGRAHKSNNIMILVDLKNEVWYQKCHDPVCKAENFKSDCFPLPAEVCL--LFLFKEE-E 477
AthPrimpol   RIGREHKSNHVMYIVDLRRGIYYQKCYDPDCRDYR--SPIRPVPDSYLPEDMVYYQGATQ 531

                                                      _____RPA-BD A__
HsaPrimpol   EFTTDEAD-ETRSNETQNPHKPSPSRLSTGASADA--VWDNGIDDAYFLEATEDAELAEA 534
AthPrimpol   NLCDNLYSEGECHVDKECD--P------DSA-ASRGSWWLEAVKVADDLESKPKTLEPLT 582

                       _____RPA-BD B___
HsaPrimpol   AENSLLSYNSE-VDEIPDE-LIIEV-LQE---------- 560
AthPrimpol   WENYEEDDDWWVAAAE-ESLKQIASL------SPSPKCP 614
```

Fig. S2 Amino acid sequence alignment of AtPrimPol with HsPrimPol. The extra N-terminal region present in plant Prim-Pols is not present in HsPrimPol. The amino acids corresponding to AEP domain are colored in orange (motif A), green (motif B) and blue (motif C). Conserved residues of AEP domain are indicated in yellow. The Zn++ finger domain is colored in pink, and the conserved cysteines and the histidine involved in Zn++ coordination are highlighted. The RPA binding domain, particularly, motif A is indicated in red and a predicted motif B binding site is also indicated.

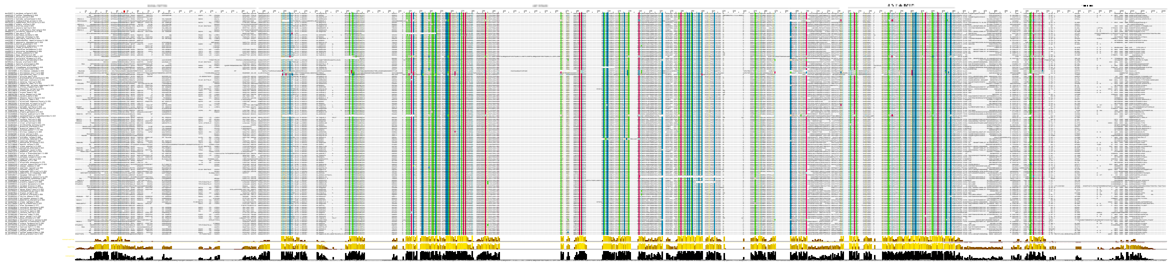

Fig. S3 Amino acid sequence alignment of plant PrimPols.

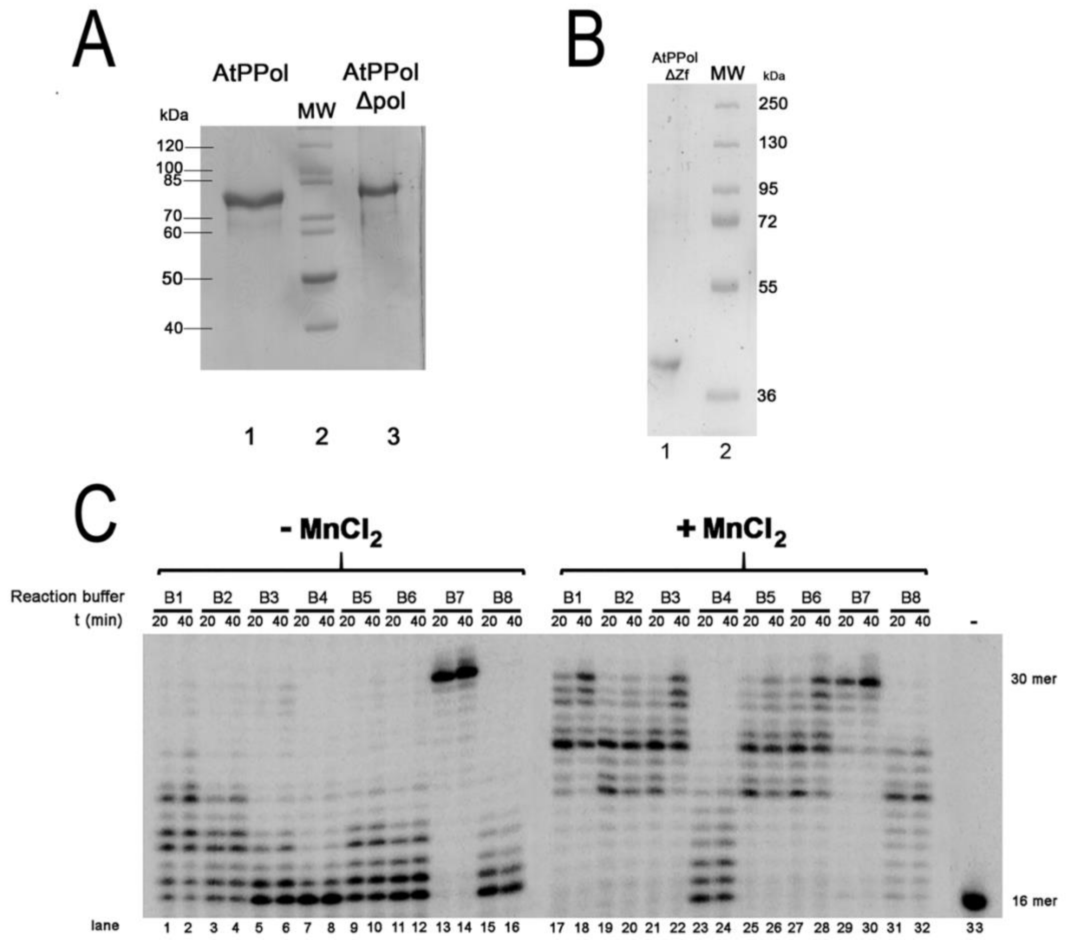

Fig. S4 Heterologous purification and enzymatic activities of AtPrimPol in diverse buffers. A and B) Purification of AtPrimPol in its variants. A) 12% SDS-PAGE Coomassie blue stained showing the final purification of wild type AtPPol, and a mutant defective in metal binding in the polymerase and therefore inactive as a DNA polymerase (AtPPol and AtPPolΔpol, respectively) B) 15% SDS-PAGE Coomassie blue stained showing the final purification of a AtPPol lacking its Zn++ binding domain (AtPPolΔZnf) **C) Polymerization by AtPrimPol using different reaction buffers.** Reactions containing 10 nM of 16-mer radiolabeled primer hybridized with 30-mer undamaged primer template were mixed with 200 nM of wild-type AtPrimPol, with or without 1 mM $MnCl_2$ in the presence of different buffers where B1 contained 10 mM Tris-HCl pH 7.5 , 10 mM MgCl2, 50 mM NaCl, 0.1 $mgmL^{-1}$ BSA; B2 is 50 mM Potassium Acetate, 20 mM Tris-acetate pH 7.9 ; B3 is 20 mM

Tris-HCl pH 8.8, 10 mM $(NH_4)_2SO_4$, 10 mM KCl, 0.1 $mgmL^{-1}$, 0.1 % (v/v) Triton X-100, 2 mM $MgSO_4$;B4 is like 1X Phusion HF reaction buffer; B5 is 33 mM Tris-acetate pH 7.9, 10 mM magnesium acetate, 66 mM potassium acetate, 0.1 $mgmL^{-1}$ BSA; B6 is 33 mM Tris-acetate pH 7.9, 10 mM Mg-acetate, 66 mM K-acetate, 0.1% (v/v) Tween 20, 1 mM DTT; B7 is 10 mM Bis-Tris-Propane-HCl pH 7, 10 mM $MgCl_2$, 1 mM DTT, and B8 is 40 mM Tris-HCl pH 8.0, 25 mM NaCl, 8 mM $MgCl_2$, 2 mM spermidine-$(HCl)_3$, 5 mM DTT. Reactions were started by addition of 200 μM dNTPs and stopped at 20 and 40 min. (**left panel**) when is used the buffer B7 a robust full polymerization extension is displayed even in absence of manganese (lane 14)

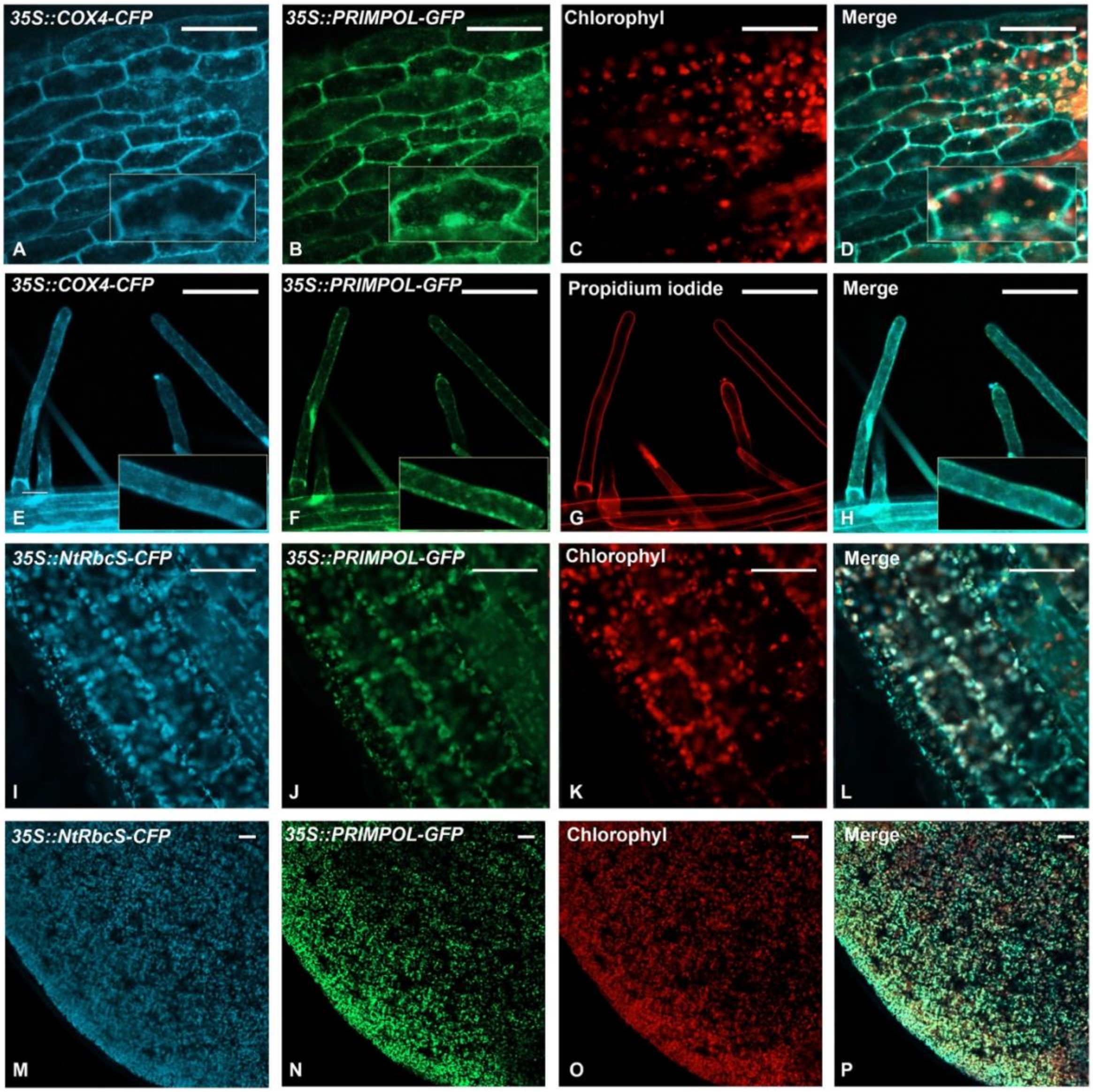

Fig. S5 Subcellular co-localization of AtPrimPol and organelle markers targeted to different compartments of Arabidopsis cells. 3 day old Arabidopsis seedlings harboring-35S::COX4-CFP as a marker for mitochondrial targeting and 35S::NrRbcS-CFP as a marker for chloroplast targeting visualized by confocal microscopy. 35S::COX4-CFP marker co-expressed in hypocotyl cells with 35S:PRIMPOL-GFP (A-D) and in root hair cells (E-H). Colocalization signal of 35S:PRIMPOL-GFP with 35S::NrRbcS-CFP occurs in hypocotyl cells (I-L) and cells of cotyledons (M-P). Bars in A to L = 50 μm. Bars in M to P = 20 μm.).

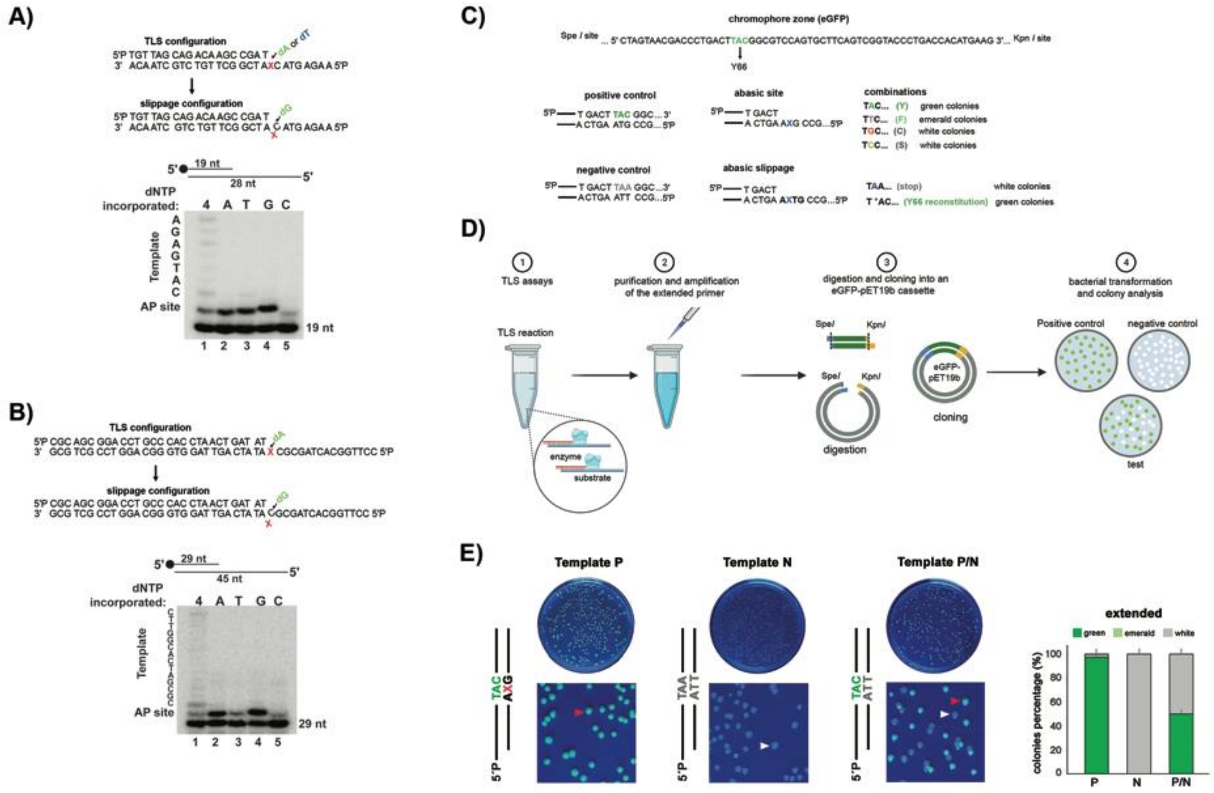

Fig. S6 Nucleotide incorporation by AtPrimPol opposite an abasic site. Nucleotide incorporation on an AP site with two different sequence contexts (A and B) using individual dNTPs. C and D) Diagram showing the rationale for the GFP based system to asses AtPrimPol replication slippage opposite an abasic site. This systems is based on a primer hybridized to a template with a modified nucleotide sequence for eGFP at an ATG codon (encoding the Tyr residue that yields the eGFP chromophore) or a a primer hybridized to a template in which the ATG codon is modified by an A(AP)G-like codon. Incorporation of dAMP would restore the Tyr codon and fluorescence would be observed. Replication slippage opposite an abasic site is assed in a parallel experiment, using a primer hybridized to a templated harboring A(AP)TG-like codon (4 bases instead of 3 bases for this codon-like sequence). In primer extension reactions in which the AP site is skipped and dAMP is incorporated, the ATG codon that codes for Tyr would be restored, and green colonies would be observed. The diagram was created using BioRender (https://biorender.com/) E) Control experiments using annealed oligonucleotide and

different sequences of the GFP marker. In the left panel, the chromophore sequence contained a TAC codon in the leading strand and an AP site denoted by X, in the center plane the annealed double stranded DNA contained a stop codon annealed with its complementary strand, and in the right panel a Tyr codon is annealed to a mismatched oligonucleotide harboring a stop codon. Red arrows indicate a colony overexpressing GFP and white arrows a colony overexpressing a non-functional GFP. The graphic shows the relative percentage between white and green colonies counted by triplicate.

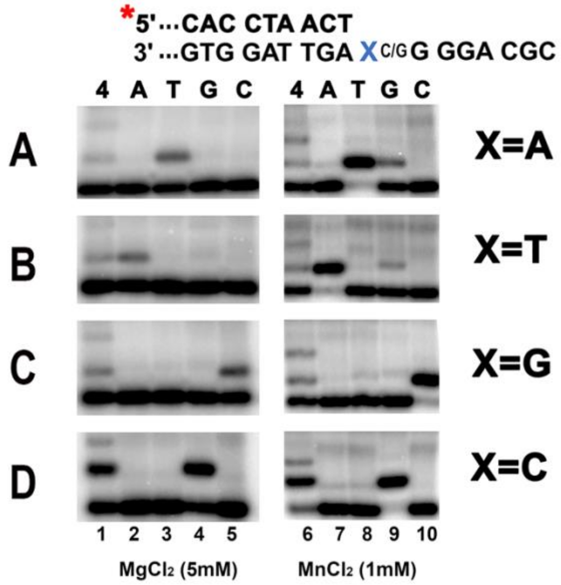

Fig. S7. Nucleotide incorporation opposite erroneous Watson-Crick pairs by AtPrimPol. Single nucleotide insertion reaction initiated by addition of 100 mM of dATP, dGTP, dTTP or dCTP. Accurate incorporation is visualized in all templates (A, T, G or C) in conditions incubated with $MnCl_2$. In contrast misincorporation products are observed when $MnCl_2$ is used.

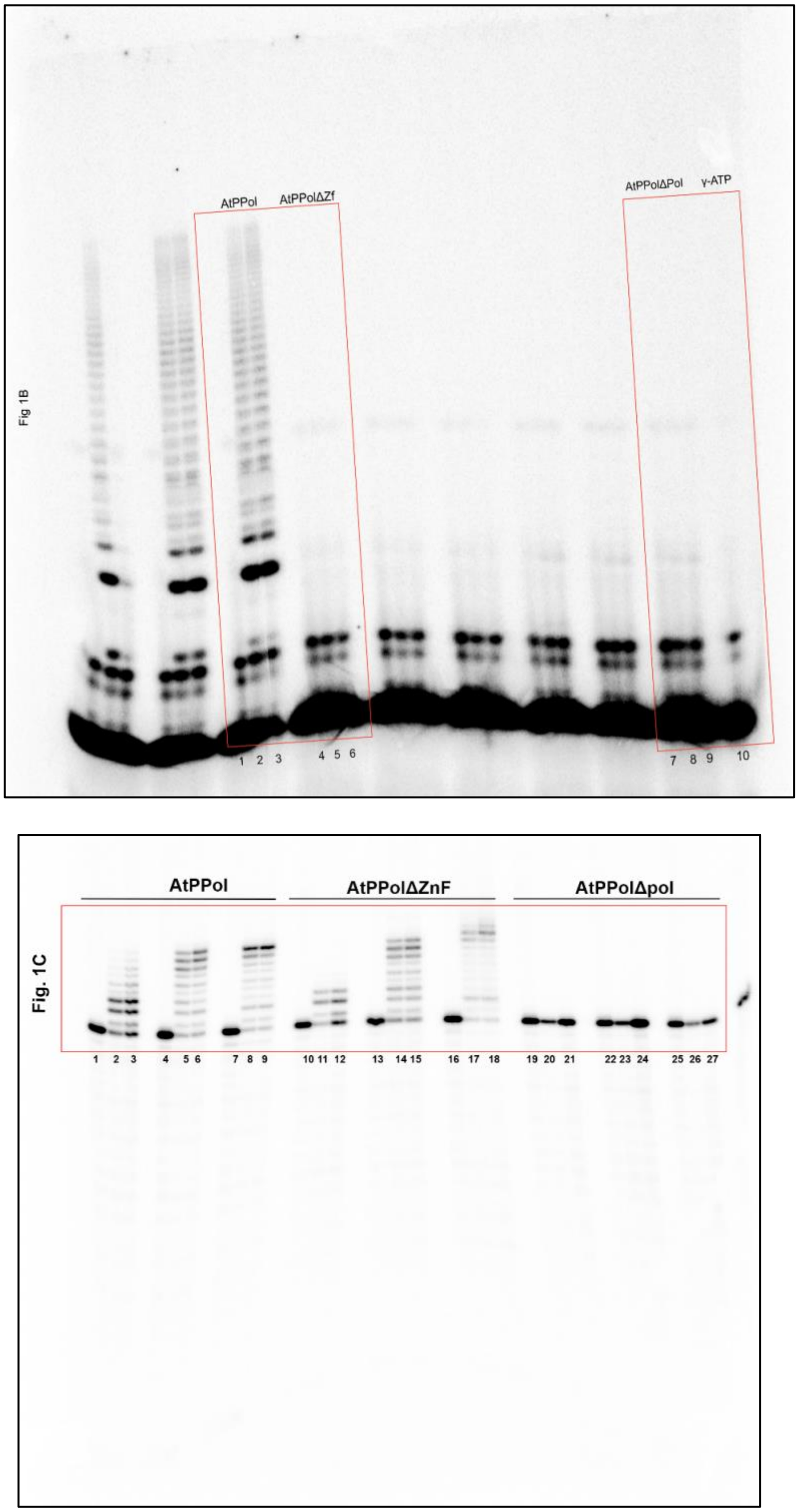

Fig. S8. Full-length gels corresponding to the experiments shown in Fig. 1. Red boxes correspond to the regions used to assemble the composite figure

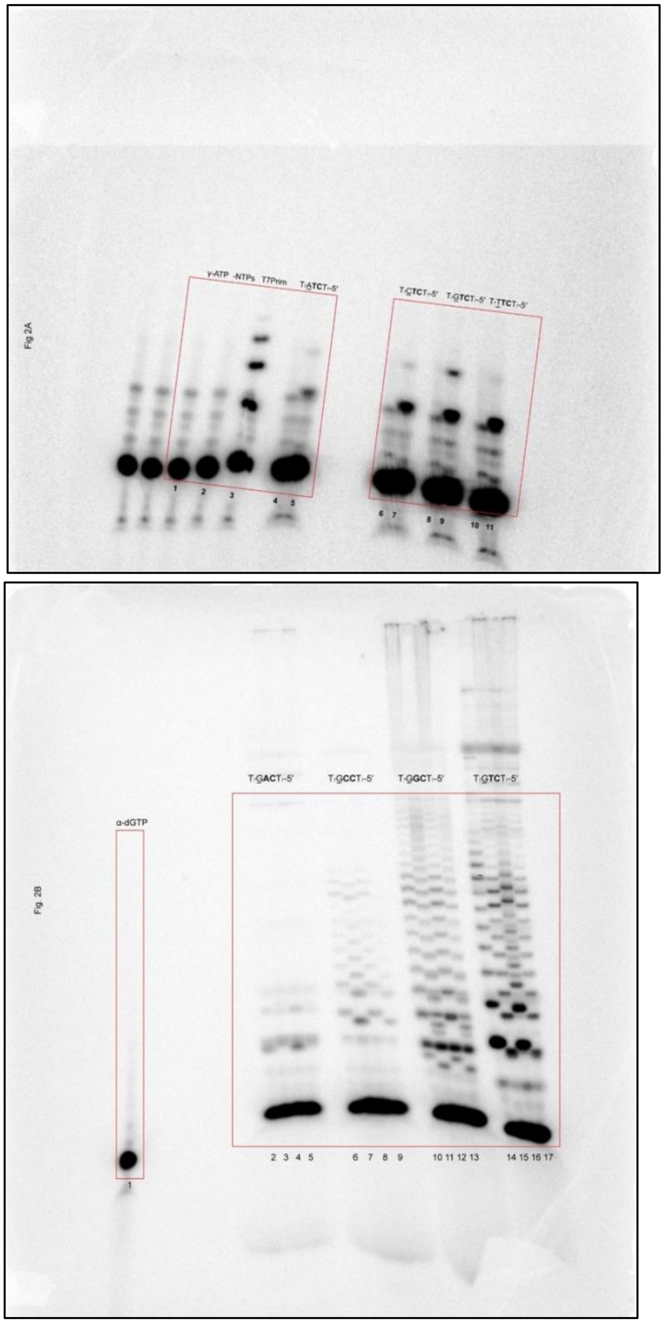

Fig. S9 Full-length gel of the experiments shown in Fig. 2A and 2B. Red boxes delimit the regions used to assemble the composite figure

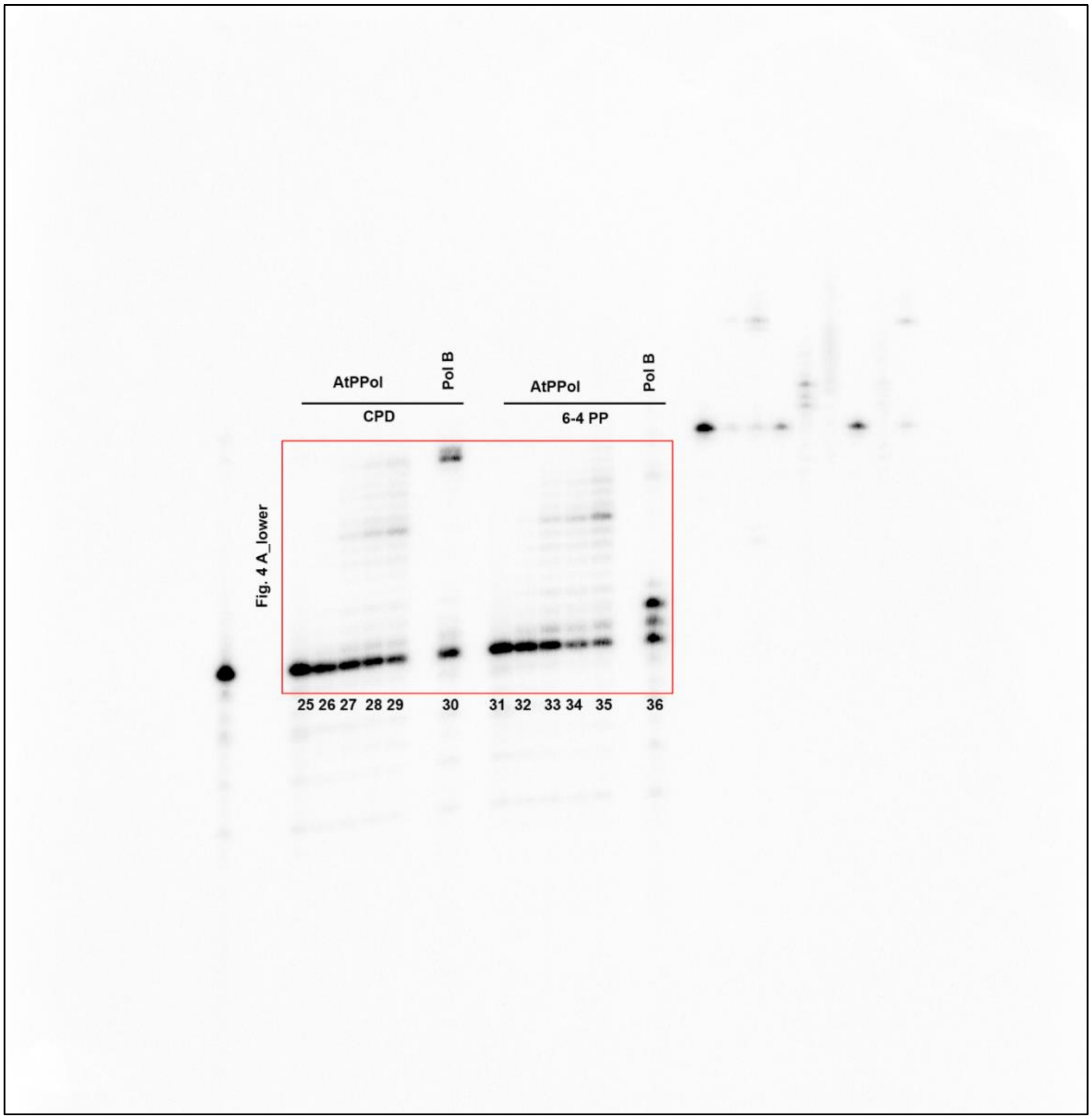
AtPPol
Pol B
AtPPol
Pol B
CPD
6-4 PP
Fig. 4 A_lower
25 26 27 28 29
30
31 32 33 34 35
36

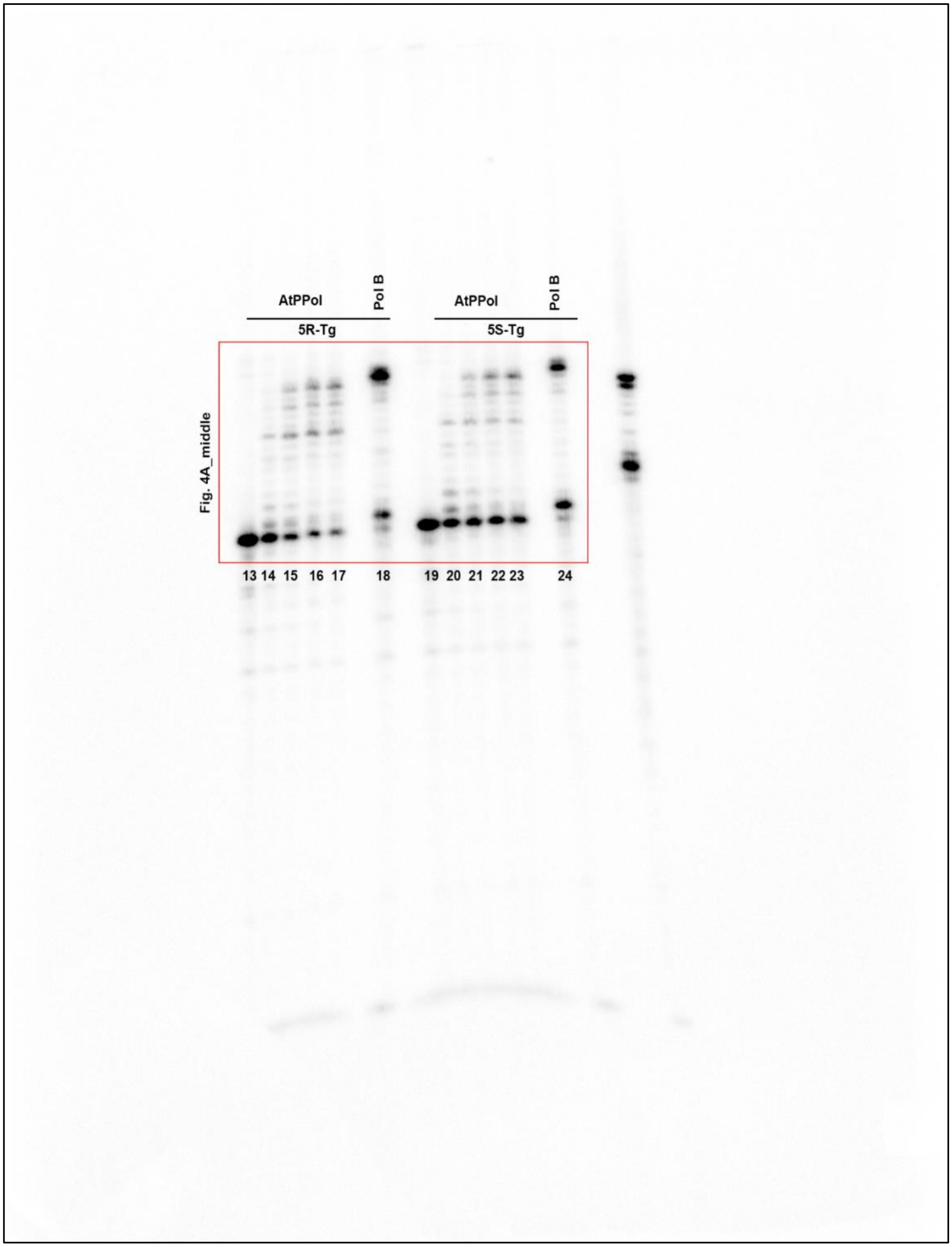

AtPPol
Pol B
5R-Tg
AtPPol
Pol B
5S-Tg
Fig. 4A_middle
13 14 15 16 17 18
19 20 21 22 23 24

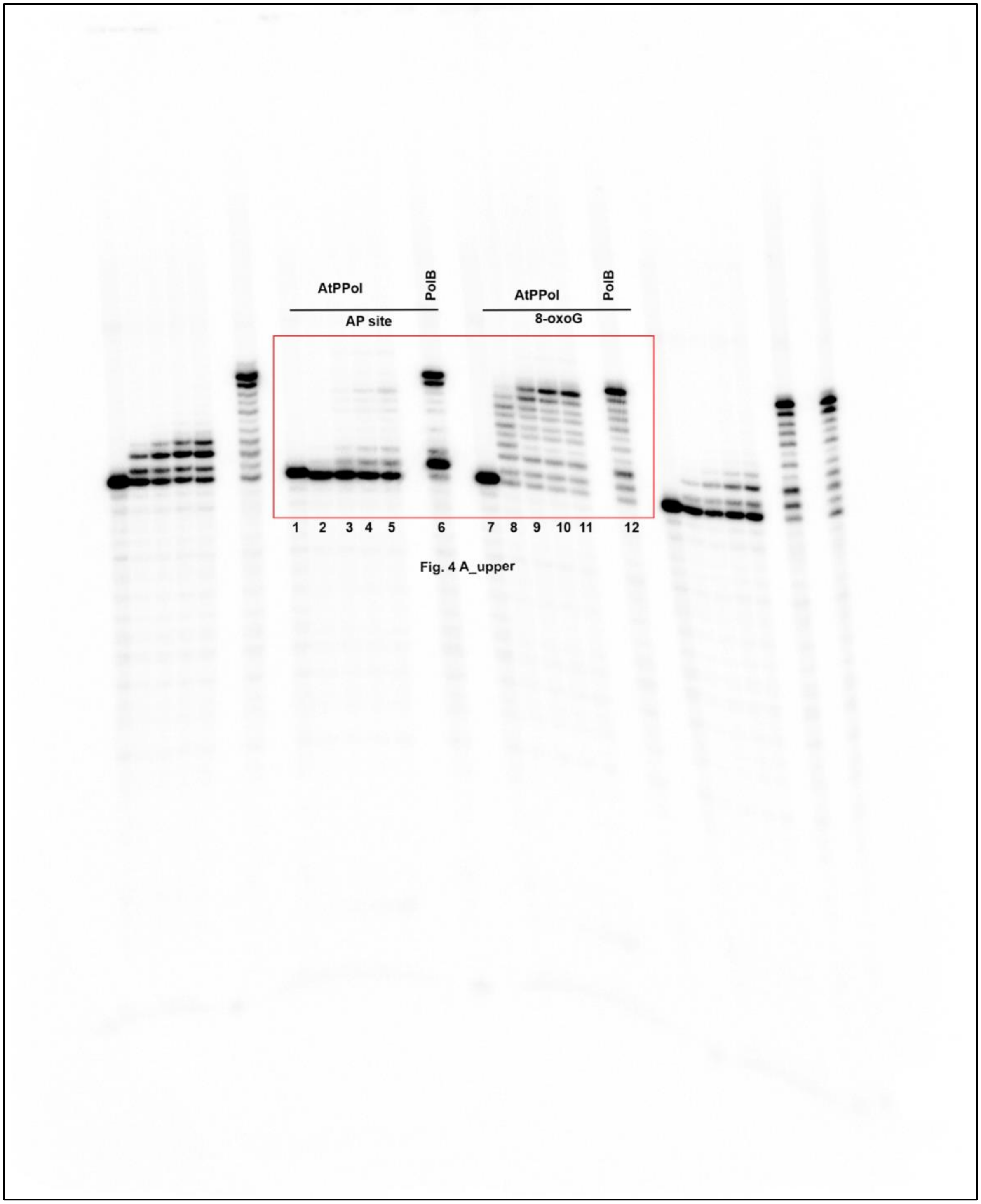

Fig. 4 A_upper

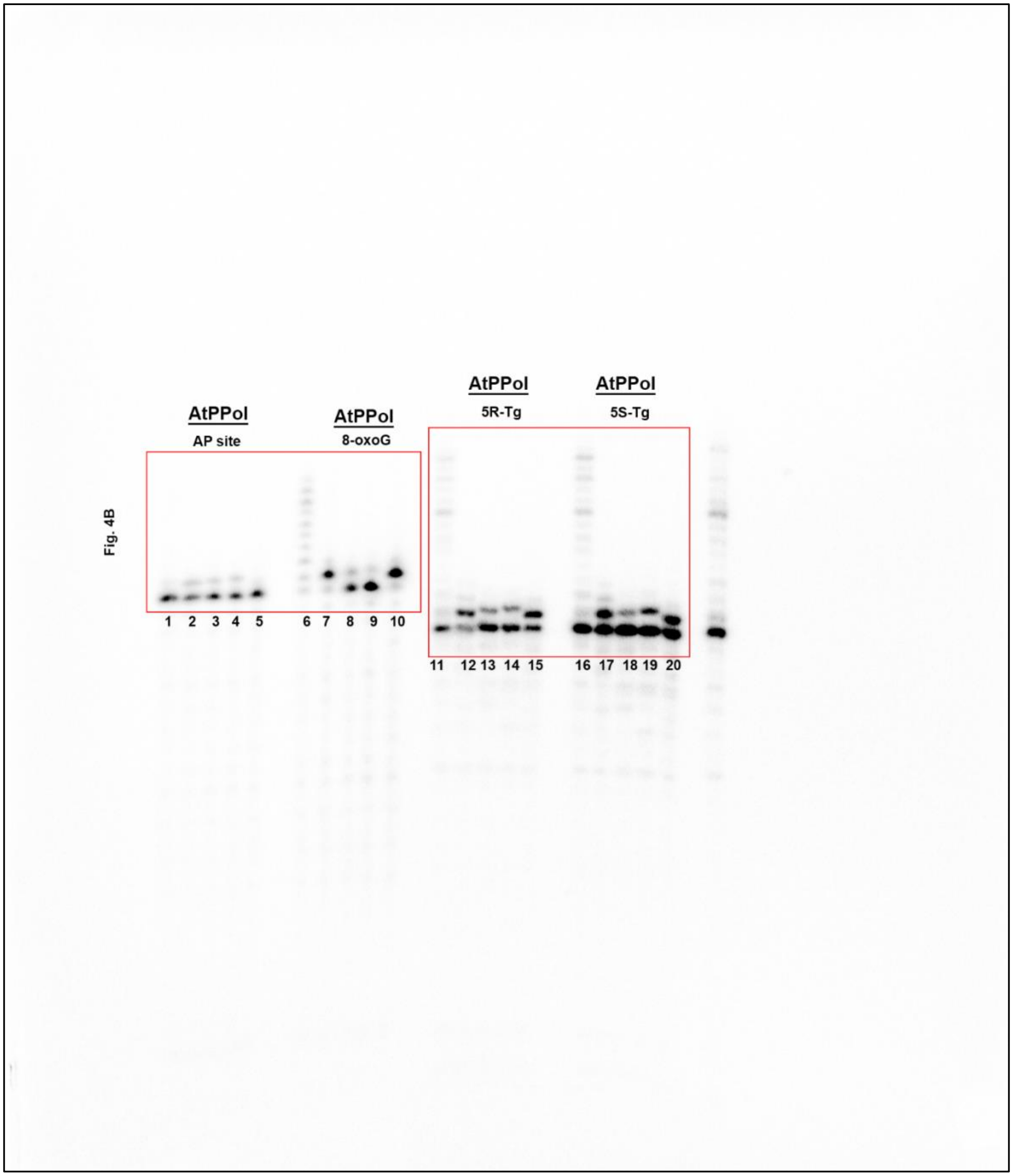

Fig. S10. Full-length gels for the experiments shown in Fig. 4A and 4B. Red boxes correspond to the regions used to form the composite figure

Fig. 5A

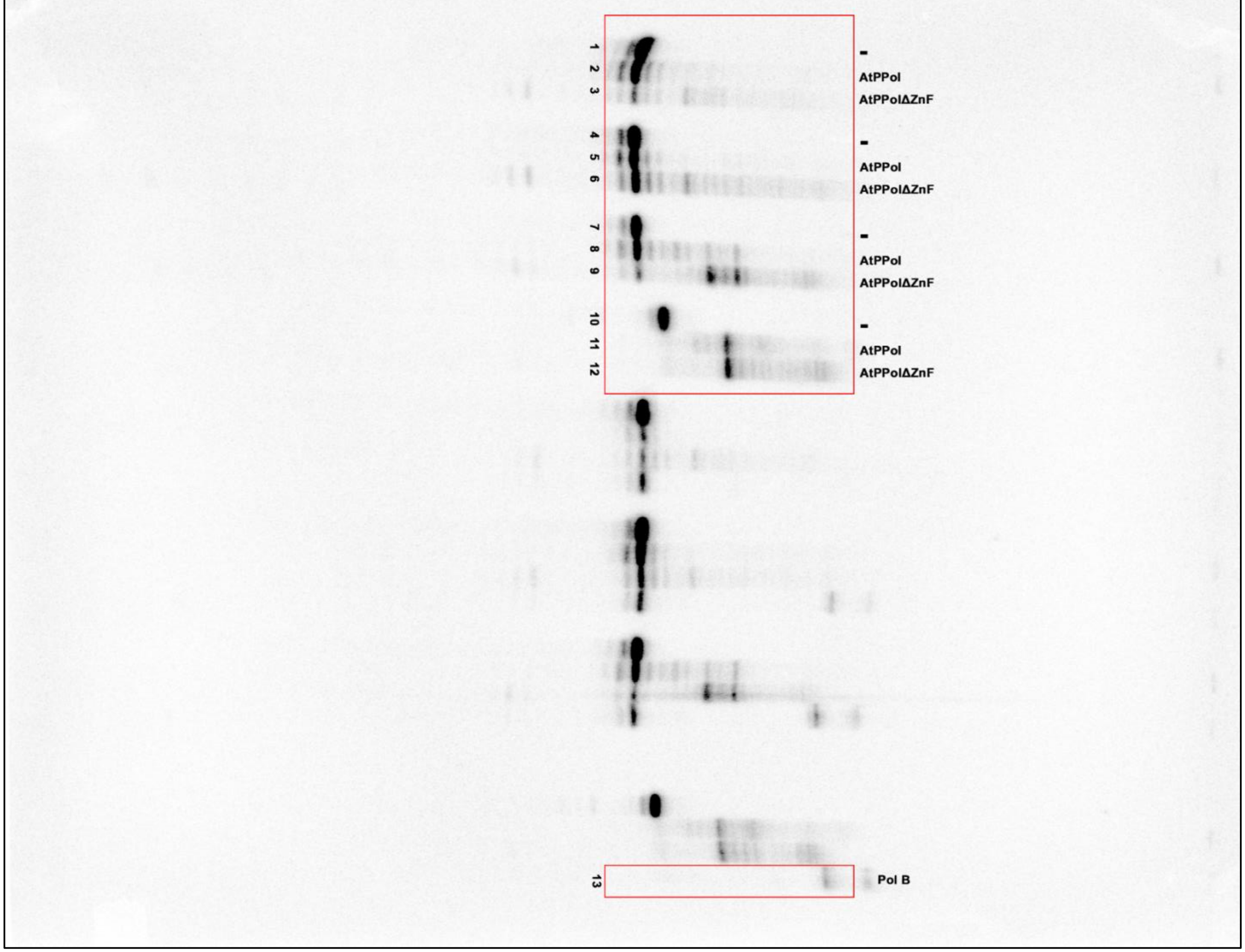
-
AtPPol
AtPPolΔZnF
-
AtPPol
AtPPolΔZnF
-
AtPPol
AtPPolΔZnF
-
AtPPol
AtPPolΔZnF
Pol B
1 2 3 4 5 6 7 8 9 10 11 12 13

Fig. 5B

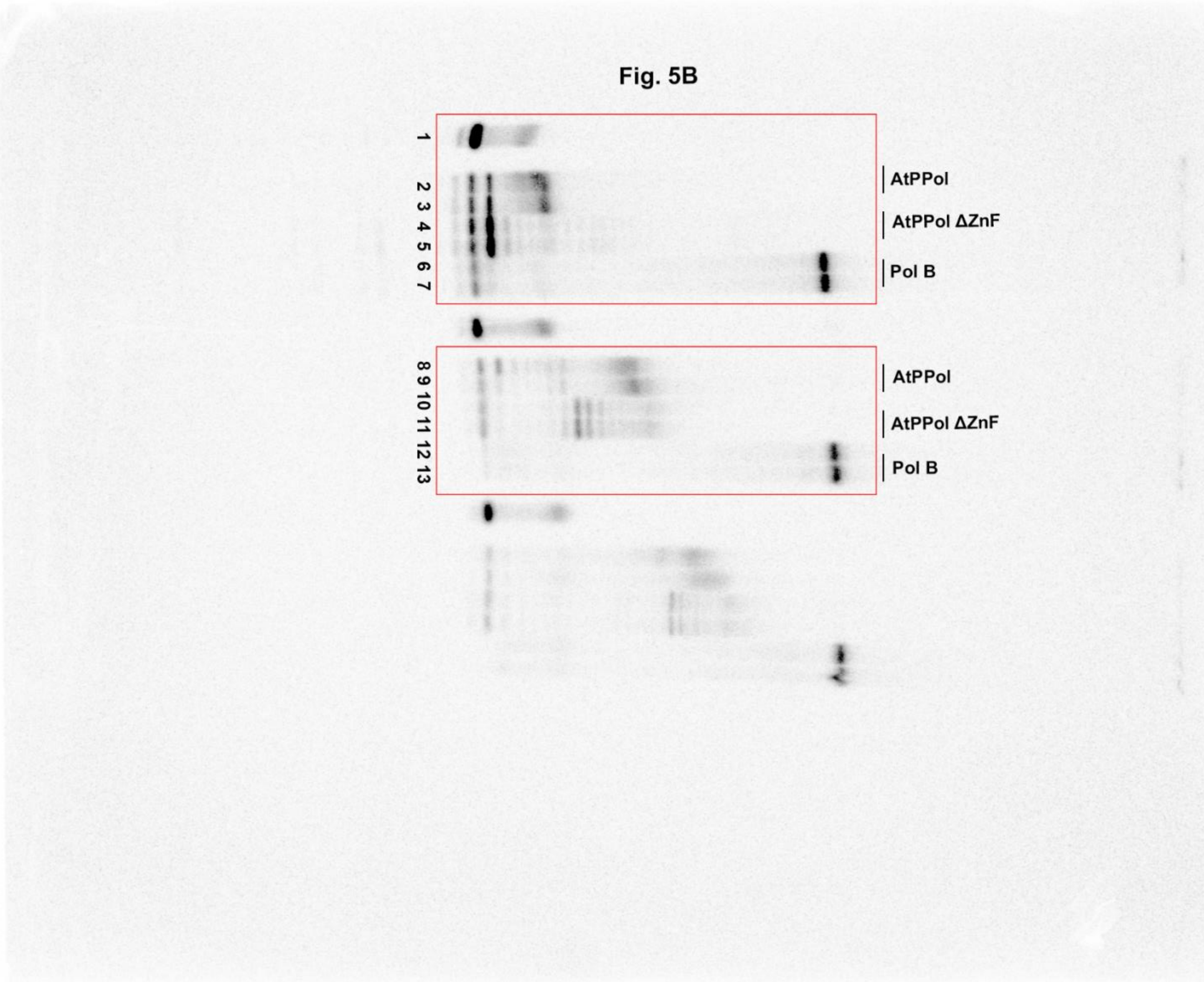
1
2 3 4 5 6 7
8 9 10 11 12 13
AtPPol
AtPPol ΔZnF
Pol B
AtPPol
AtPPol ΔZnF
Pol B

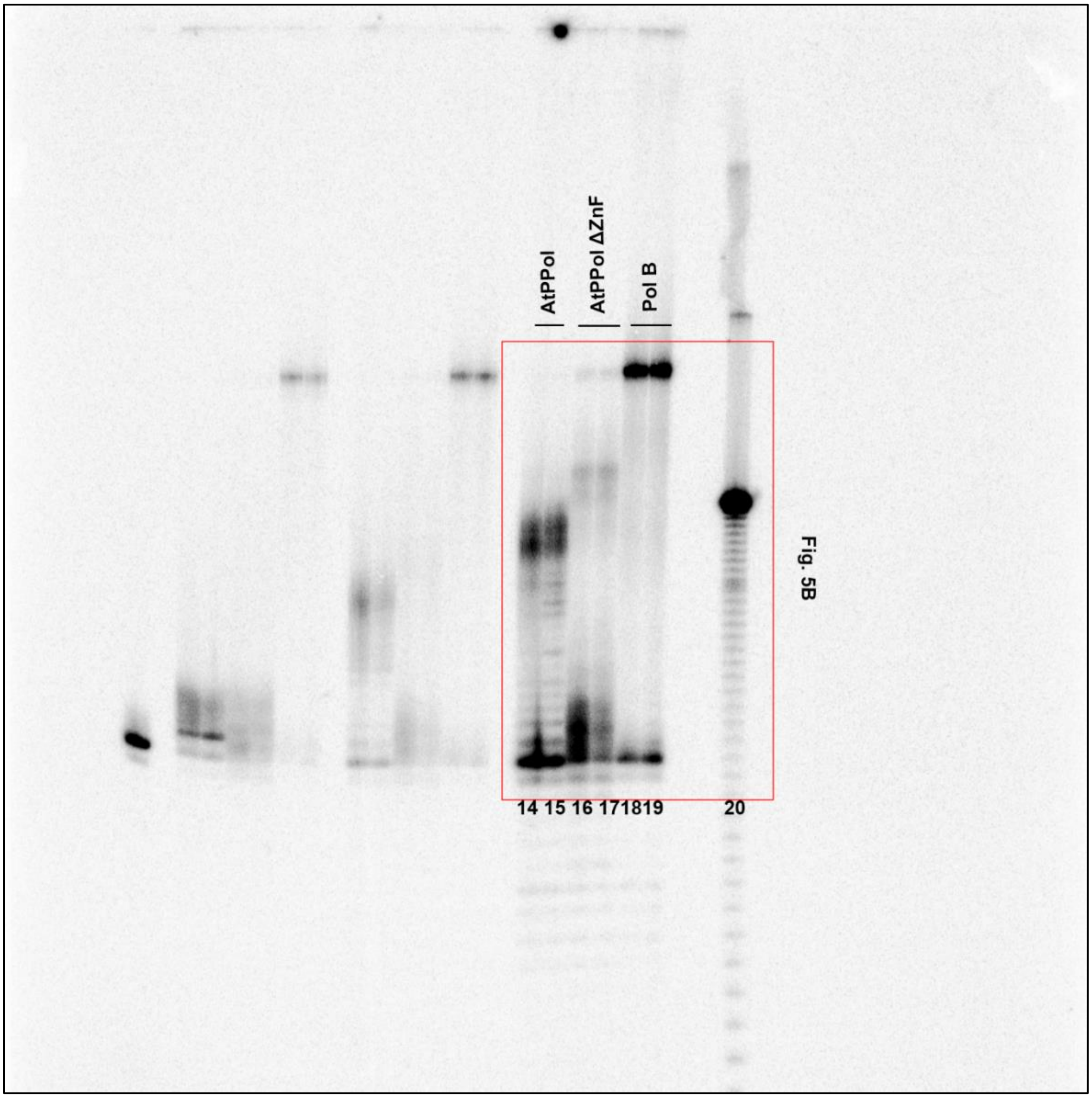

Fig. 5B

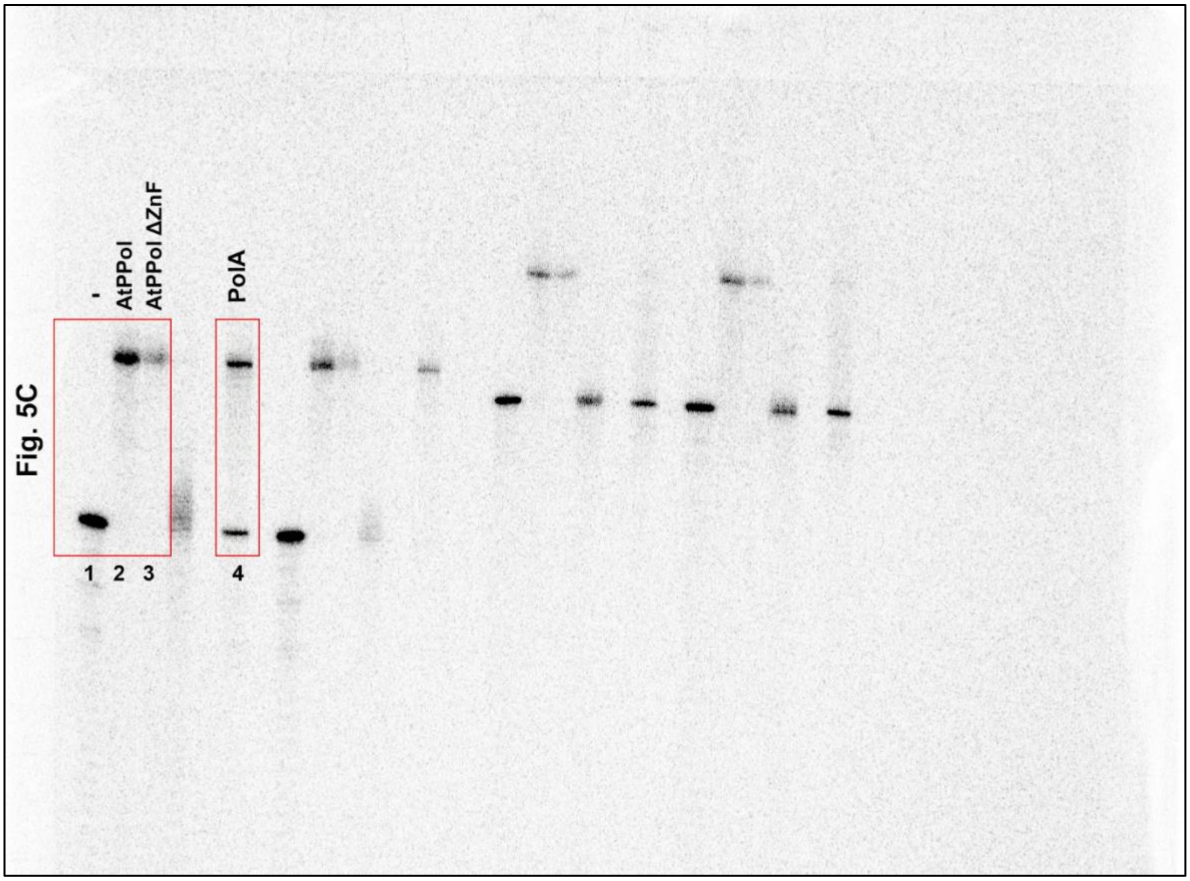

Fig. 5C
-
AtPPol
AtPPol ΔZnF
PolA
1 2 3 4

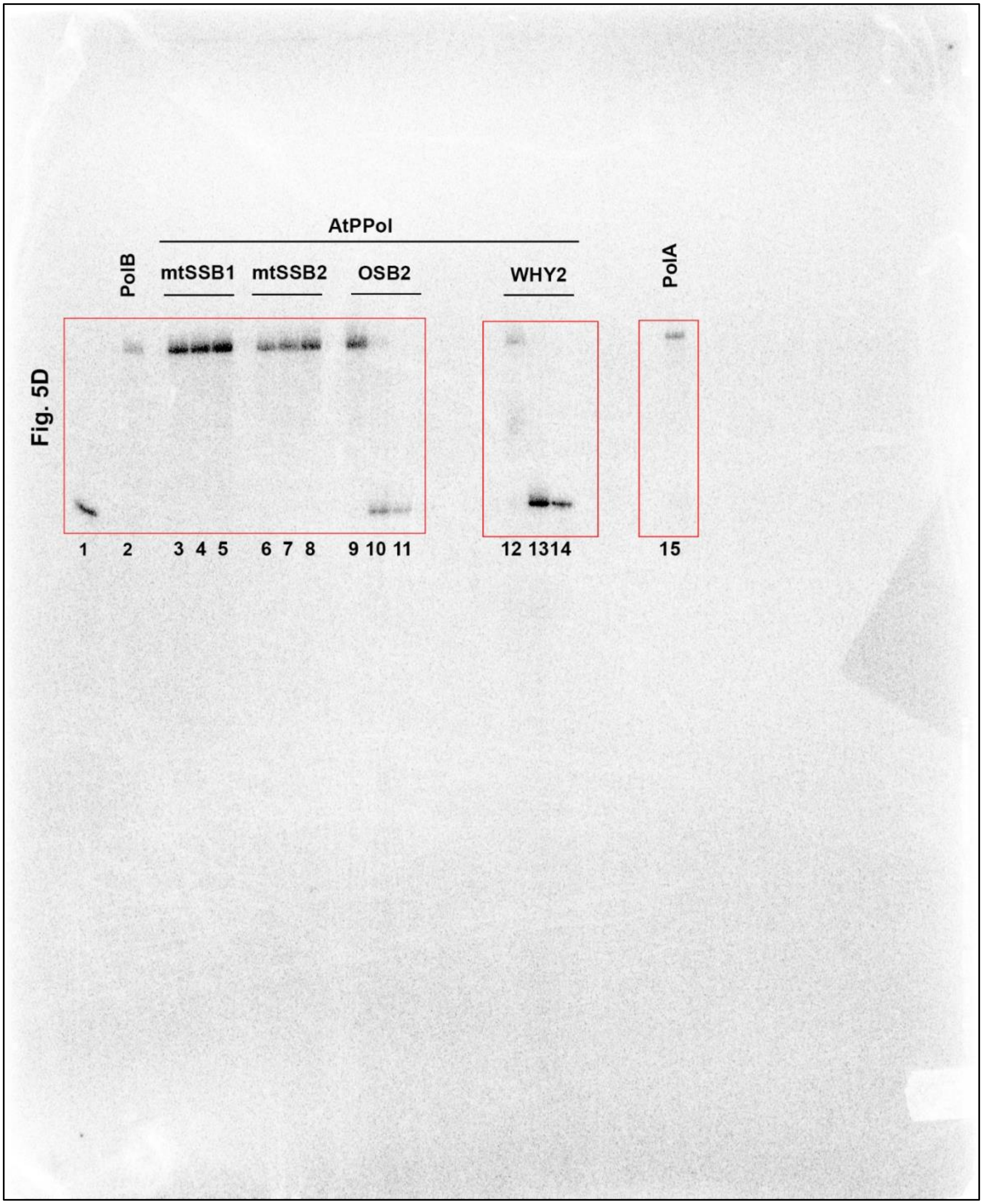

Fig. S11. Full-length gels used to assemble the experiments shown in Fig. 5. Red boxes correspond to the regions used to form the composite figure.

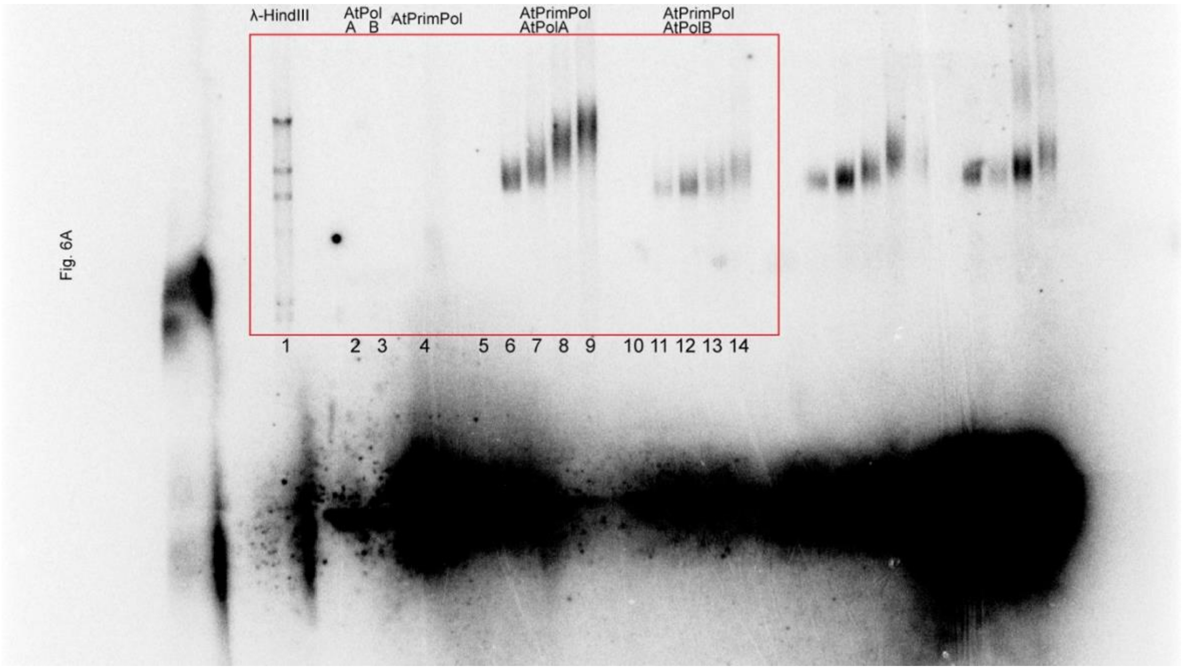

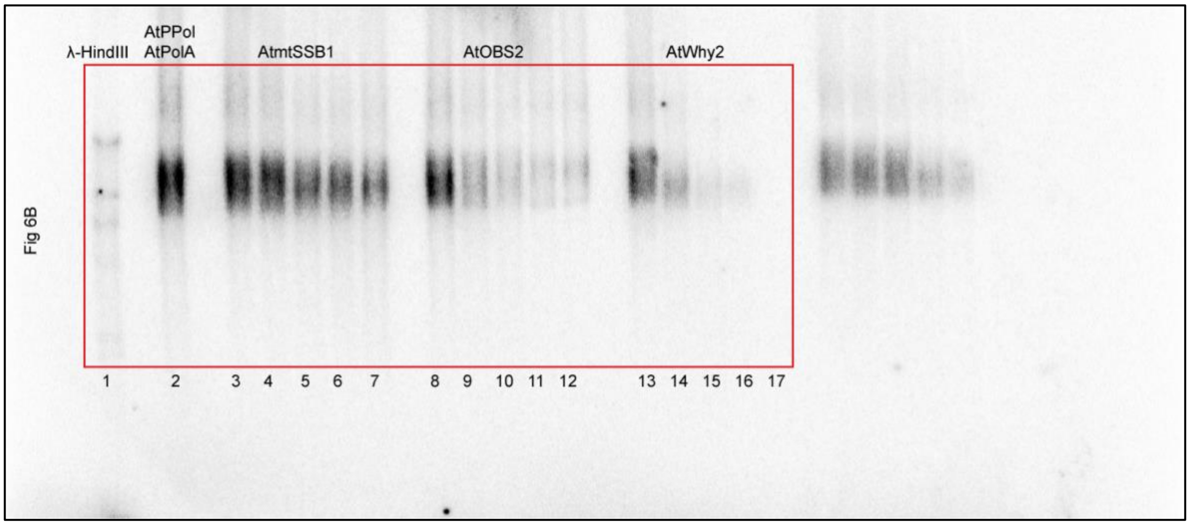

Fig. S12. Full-length gels of experiments shown in Fig. 6A and 6B. Red boxes correspond to the regions used to form the composite figure.

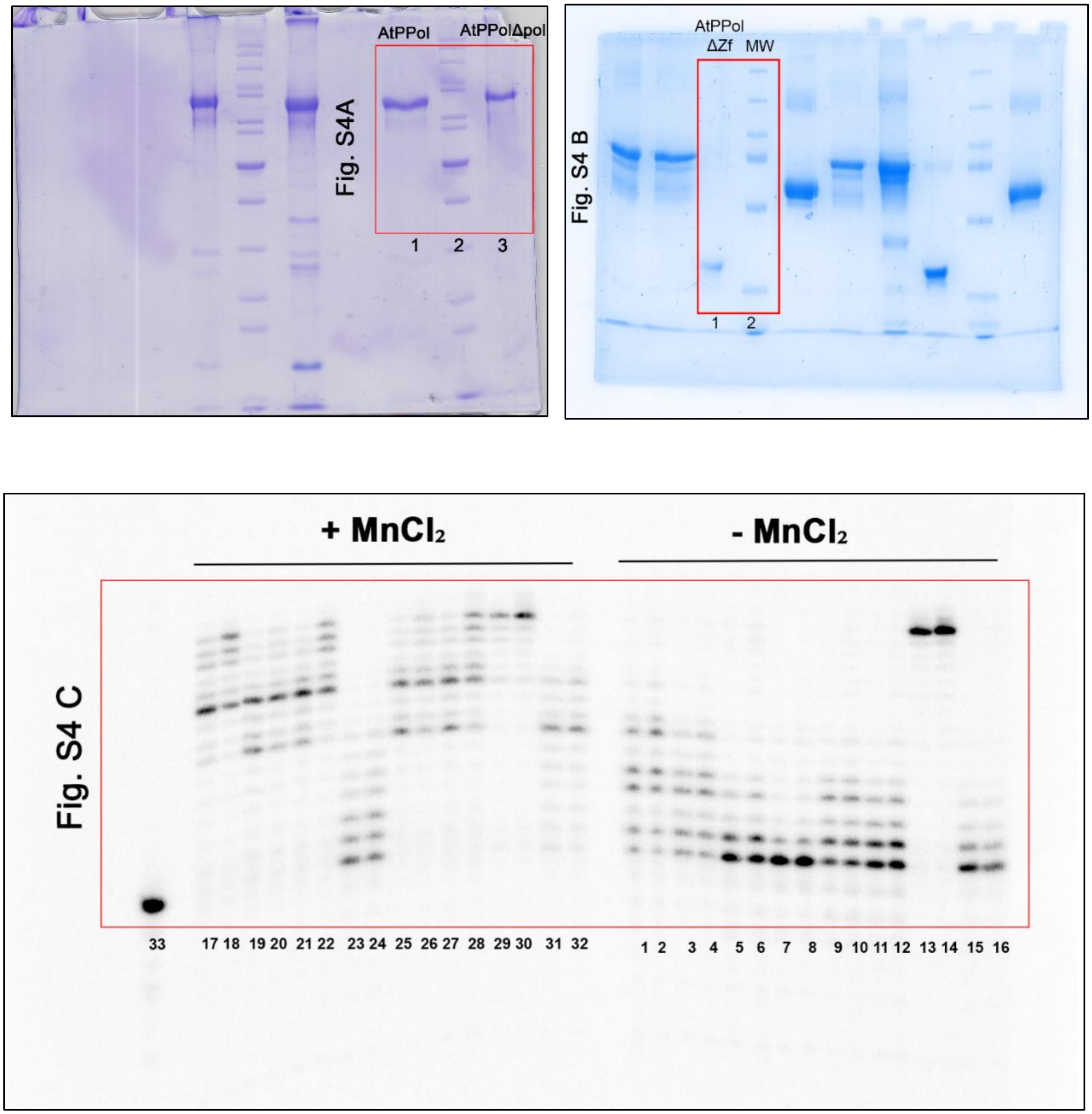

Fig. S13. Full-length gels of experiments shown in Fig S4. Red boxes correspond to the regions used to assemble the composite figure.

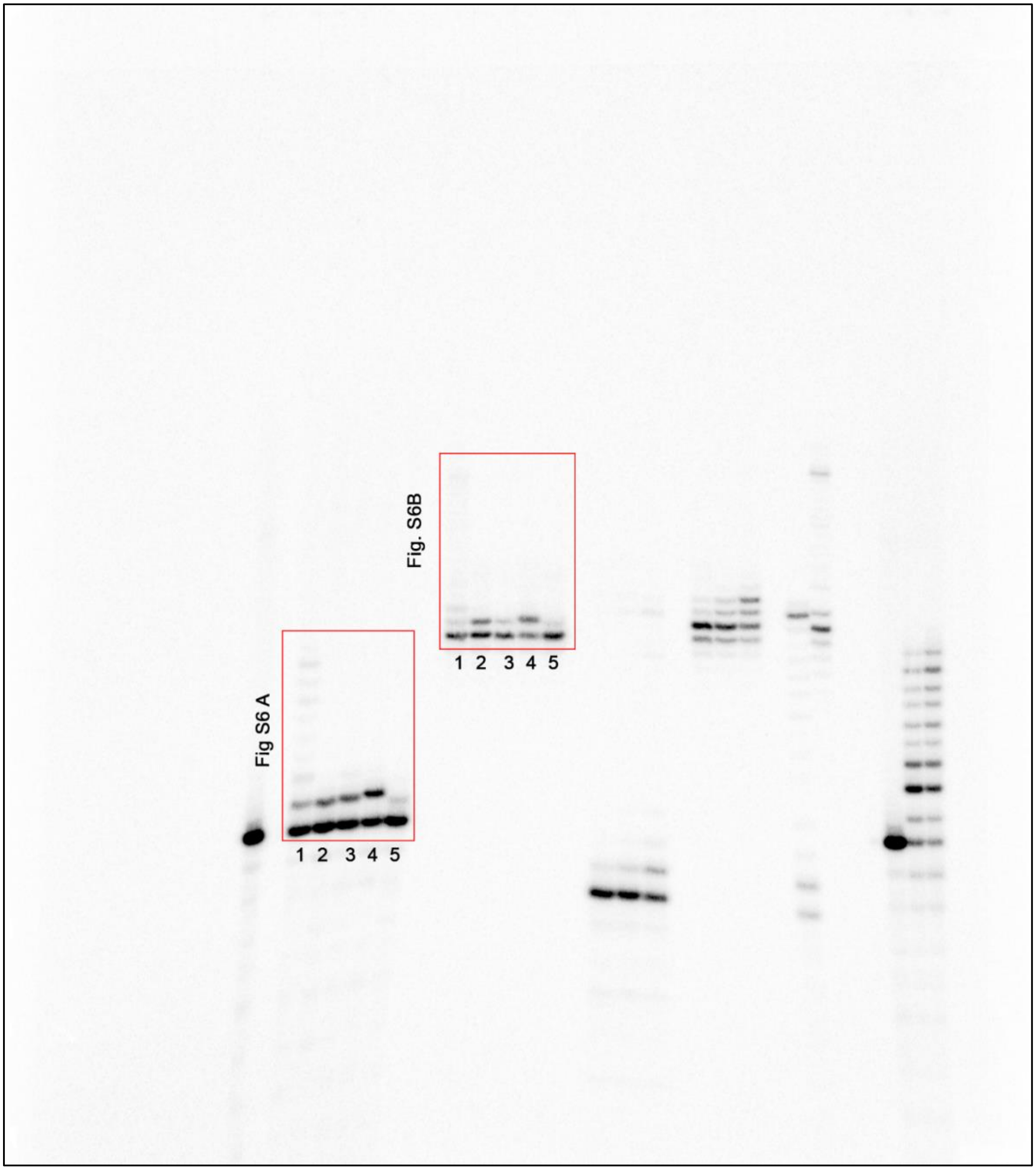

Fig. S14. Full-length gels of experiments shown in Fig S6. Red boxes correspond to the regions used to assemble the composite figure.

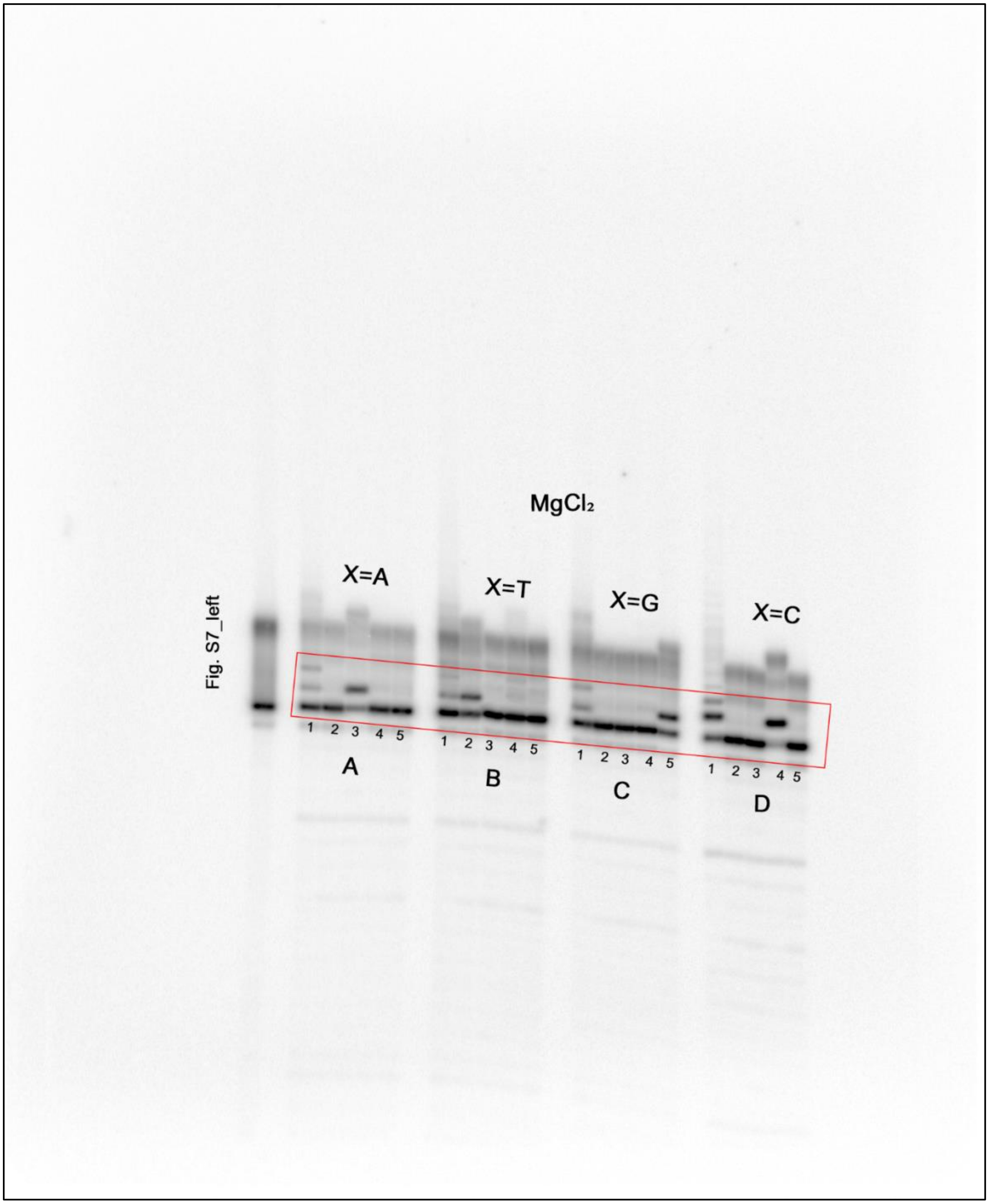
Fig. S7_left
MgCl₂
X=A
X=T
X=G
X=C
1 2 3 4 5
1 2 3 4 5
1 2 3 4 5
1 2 3 4 5
A
B
C
D

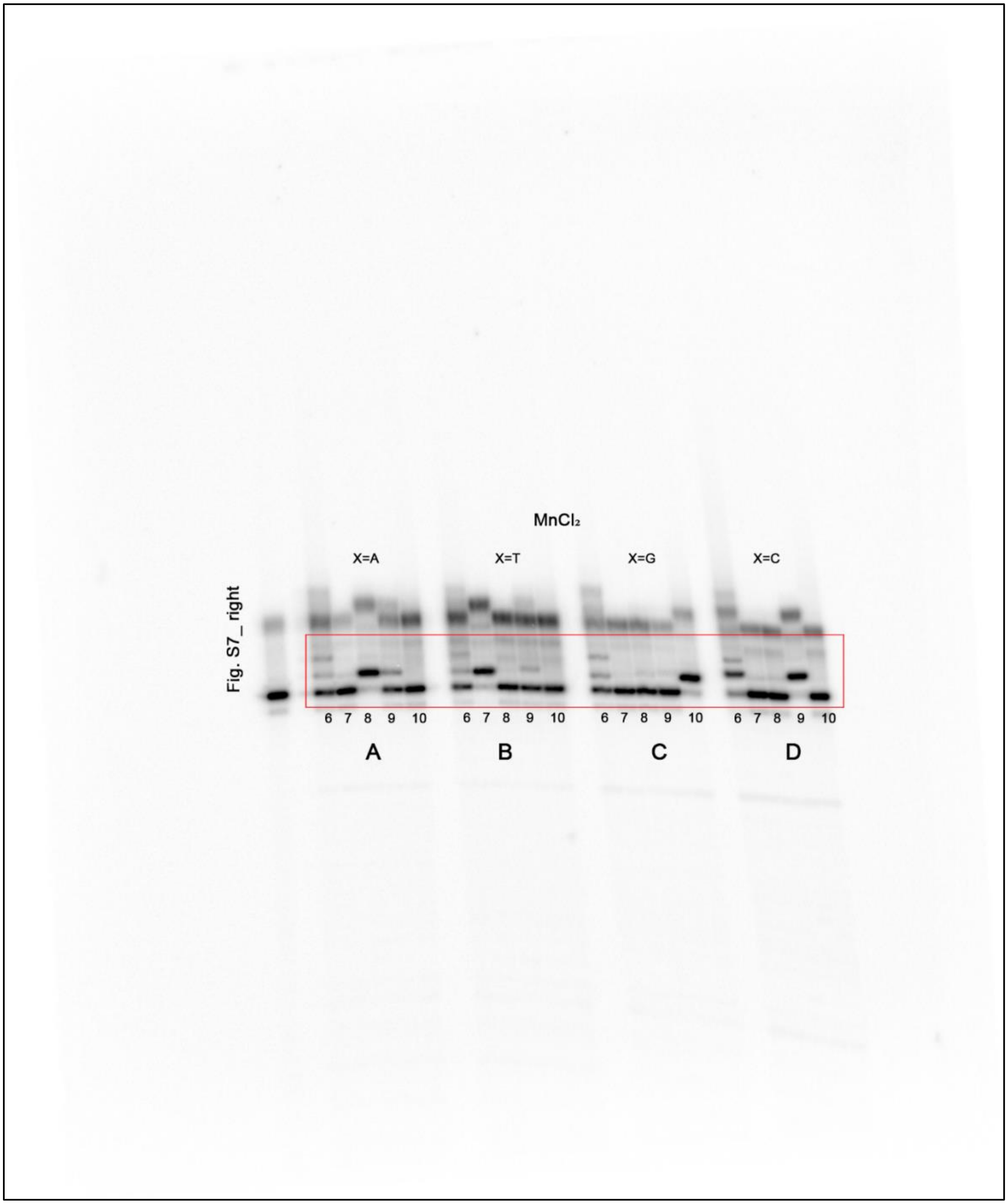

Fig. S15. Full-length gels of experiments shown in Fig S7. Red boxes correspond to the regions used to assemble the composite figure
